# Essential oils and plant extracts for tropical fruits protection: From farm to table

**DOI:** 10.3389/fpls.2022.999270

**Published:** 2022-09-29

**Authors:** Nur Aisyah Mohd Israfi, Muhamad Israq Amir Mohd Ali, Sivakumar Manickam, Xun Sun, Bey Hing Goh, Siah Ying Tang, Norsharina Ismail, Ahmad Faizal Abdull Razis, Soo Ee Ch’ng, Kim Wei Chan

**Affiliations:** ^1^ Natural Medicines and Products Research Laboratory, Institute of Bioscience, Universiti Putra Malaysia, Serdang, Selangor Darul Ehsan, Malaysia; ^2^ Department of Biomedical Sciences, Faculty of Medicine and Health Sciences, Universiti Putra Malaysia, Serdang, Selangor Darul Ehsan, Malaysia; ^3^ Chemical Engineering Discipline, School of Engineering, Monash University Malaysia, Subang Jaya, Malaysia; ^4^ School of Energy and Chemical Engineering, Xiamen University Malaysia, Sepang, Selangor Darul Ehsan, Malaysia; ^5^ Petroleum and Chemical Engineering, Faculty of Engineering, Universiti Teknologi Brunei, Bandar Seri Begawan, Brunei; ^6^ Key Laboratory of High Efficiency and Clean Mechanical Manufacture, Ministry of Education, School of Mechanical Engineering, Shandong University, Jinan, China; ^7^ National Demonstration Centre for Experimental Mechanical Engineering Education, Shandong University, Jinan, China; ^8^ Biofunctional Molecule Exploratory Research Group, School of Pharmacy, Monash University Malaysia, Subang Jaya, Malaysia; ^9^ College of Pharmaceutical Sciences, Zhejiang University, Hangzhou, China; ^10^ Tropical Medicine and Biology Platform, School of Science, Monash University Malaysia, Subang Jaya, Malaysia; ^11^ Department of Food Science, Faculty of Food Science and Technology, Universiti Putra Malaysia, Serdang, Selangor Darul Ehsan, Malaysia; ^12^ Laboratory of Food Security and Food Integrity (FOSFI), Institute of Tropical Agriculture and Food Security, Universiti Putra Malaysia, Serdang, Selangor Darul Ehsan, Malaysia; ^13^ CAIQTEST Malaysia Sdn. Bhd., Shah Alam, Selangor, Malaysia

**Keywords:** essential oils, plant extracts, tropical fruits, plant diseases, protection, biopesticides

## Abstract

The tropical fruit industry in Malaysia makes up a large proportion of the agriculture sector, contributing to the local economy. Due to their high sugar and water content, tropical fruits are prone to pathogenic infections, providing optimal microorganism growth conditions. As one of the largest exporters of these fruits globally, following other Southeast Asian countries such as Thailand, Indonesia and the Philippines, the quality control of exported goods is of great interest to farmers and entrepreneurs. Traditional methods of managing diseases in fruits depend on chemical pesticides, which have attracted much negative perception due to their questionable safety. Therefore, the use of natural products as organic pesticides has been considered a generally safer alternative. The extracts of aromatic plants, known as essential oils or plant extracts, have garnered much interest, especially in Asian regions, due to their historical use in traditional medicine. In addition, the presence of antimicrobial compounds further advocates the assessment of these extracts for use in crop disease prevention and control. Herein, we reviewed the current developments and understanding of the use of essential oils and plant extracts in crop disease management, mainly focusing on tropical fruits. Studies reviewed suggest that essential oils and plant extracts can be effective at preventing fungal and bacterial infections, as well as controlling crop disease progression at the pre and postharvest stages of the tropical fruit supply chain. Positive results from edible coatings and as juice preservatives formulated with essential oils and plant extracts also point towards the potential for commercial use in the industry as more chemically safe and environmentally friendly biopesticides.

## Introduction

In 2020, the agriculture industry recorded a contribution of 7.4% of Malaysia’s Gross Domestic Product (GDP) (Mahidin, 2021). Following rubber, oil palm and paddy, tropical fruits make up a large proportion of the agriculture landscape in Malaysia (Abu Dardak, 2019). It is estimated that roughly 192,000 hectares of agricultural land in Malaysia are used to cultivate tropical fruits. Fruit export in Malaysia is valued at RM1.46 billion (USD 347 million) in 2020, making it one of the most important exported products in the agriculture sector (Abu Dardak, 2022). The fruits exported from Malaysia include seasonal fruits such as durian, rambutan, mango and mangosteen, and those grown all year round like papayas, watermelon and bananas (Arope, 1992). Among the fruits exported, bananas, pineapples, and watermelon make up the majority, with production exceeding 200 metric tons per year (Rozana et al., 2017).

Tropical fruits are prone to diseases such as anthracnose, rotting and mould. Currently, Malaysia’s conventional way of managing and treating these diseases is concentrated on the use of chemical pesticides, with more than 50% of farmers preferring this approach instead of other alternatives (Chang, 2021). The inclination for farmers to opt for chemical means may be attributed to their effectiveness and accessibility (Sharifzadeh et al., 2018). However, pesticide residue is increasingly becoming a major safety concern after recorded chemical poisoning and environmental pollution cases. For example, the commonly used pesticides such as propiconazole and various organophosphorus pesticides were found to wind up in Malaysian rivers (Wee et al., 2016; Elfikrie et al., 2020). The loosely regulated use of chemical pesticides thus can pose as threats to the local communities. As a result, research has been actively looking into using nature-derived compounds as alternative organic pesticides.

**Figure 1:**
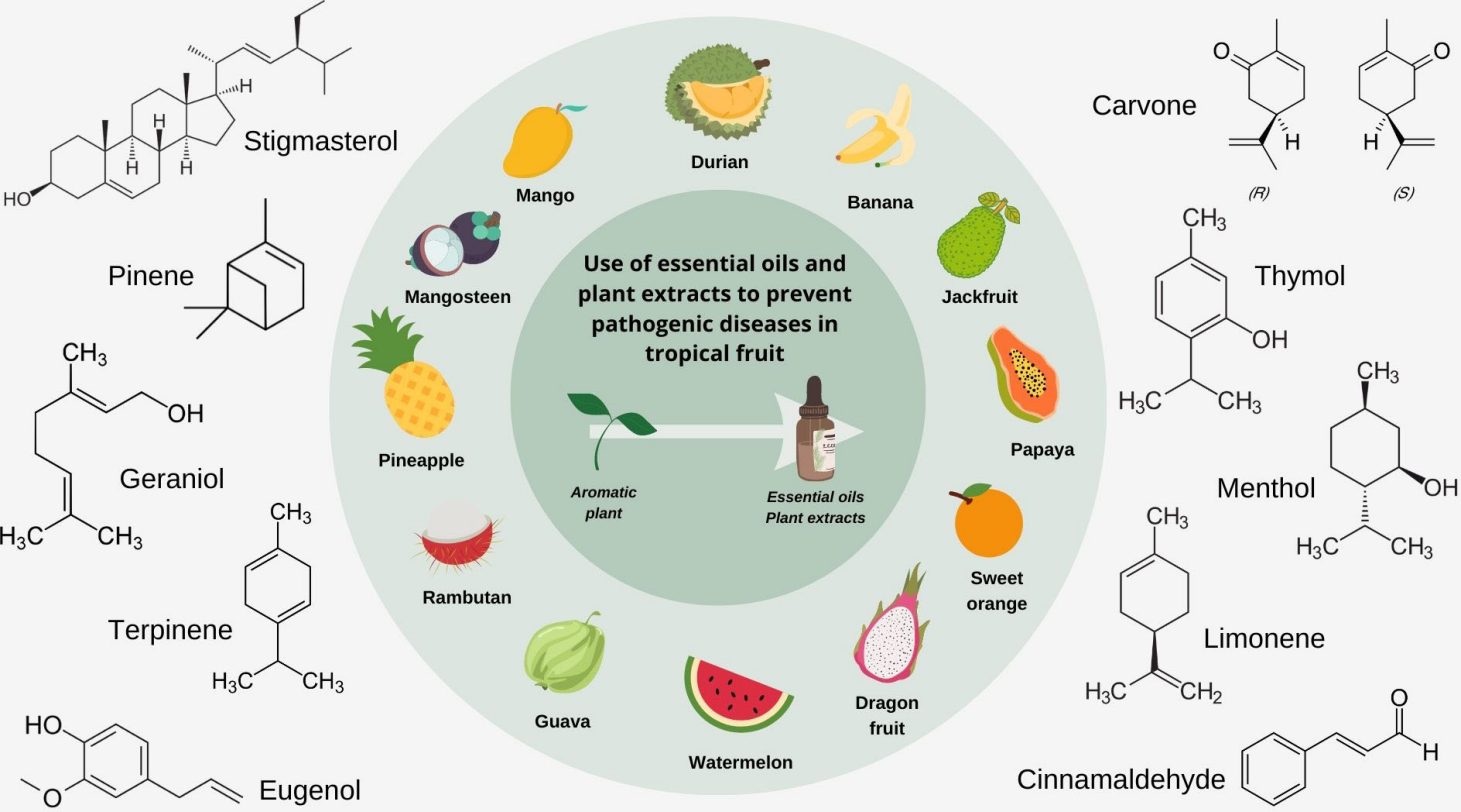
Selected tropical fruits and bioactive compounds present in essential oils and plant extracts responsible for observed antimicrobial properties.

Essential oils are obtained from distilling aromatic plants and have been gaining interest for their use in aromatherapy. Plant extracts have traditionally been used as flavoring agents and fragrances throughout history. Essential oils often contain bioactive constituents such as esters, terpenes, phenols, aldehydes and ketones, which possess antimicrobial activity (Pauli, 2001). They are relatively safer than commonly used chemical pesticides (Moharramipour and Negahban, 2014; Bhavaniramya et al., 2019; Singh et al., 2019); hence, research has been focused on understanding how the antimicrobial properties of essential oils and plant extracts can be utilized in agriculture as organic biopesticides (Lahlali et al., 2022). Therefore, this review aims to cover the literature on using essential oils and plant extracts as potential pesticides, focusing on plant diseases caused by fungi and bacteria. Bioactive compounds believed to contribute to the antimicrobial activity of essential oils and plant extracts in the management of crops are also reviewed (**Figure 1**). The review will also summarize how they can be used at different points in the supply chain of getting tropical fruits from the farm to consumers pre and postharvest stages, including processed products such as fresh-cut fruit and fruit juices.

## Tropical fruit production in Asian countries and Malaysia

Asian countries have been the main producers and exporters of tropical fruits globally, especially in the European market. Tropical fruits such as banana, mango and pineapple are among the largest cultivated and can be widely found in international markets. Meanwhile, more seasonal tropical fruits such as durian, guava, jackfruit, and mangosteen have lower cultivation and trading activities. According to the Food and Agriculture Organisation of the United Nations, FAO (2021), 2.2 million tons of mangoes were globally exported in 2020, increasing by 2.9% compared to 2019. Similarly, global papaya exports reached 353,000 tons, increasing by 2.7% from 2019. However, global exports of pineapple decreased to 3.1 million tons, representing an 8.2% fall from 2019. The decrease may be due to COVID-19 constraints that negatively impacted the global market in early 2020. Despite the fall in exportation rates, pineapple still recorded the highest global export among the three most cultivated tropical fruits.

The FAO (2021) also reported the gross exportation of tropical fruits in Asian countries from 2018 to 2019. As the largest exporter of mangoes, mangosteen and guava, Thailand recorded 260,100 tonnes of exports in 2018, which increased to 479,600 tonnes in 2019. Meanwhile, India exported 153,300 tonnes in 2018, decreasing to 147,200 tonnes in 2019. Since the fall was recorded before the COVID-19 pandemic took off, the decrease in exportation might be due to postharvest diseases, resulting in the loss of quality of fruits to be exported (Jat et al., 2020). The Philippines is the largest pineapple exporter, with exports reaching 442,100 tonnes in 2018. This number increased to 625,500 tonnes in 2019, while Malaysia exported 19,600 tonnes in 2018, reducing to 17,900 tonnes in 2019. India was the biggest exporter of papaya in 2018 at 18,000 tonnes which further increased in 2019 to 19,000 tonnes, followed by China and Malaysia.

Tropical fruits are important for Malaysia as they are the major source of local income. Over the years, the efforts to produce tropical fruits in Malaysia have been elevated to accommodate an increase in demand in the global market, contributing to the higher revenue recorded from tropical fruit trading activities. As shown in **Table 1**, Malaysia produced more than 1.5 million tonnes of fruits valued at close to RM 10 million (USD 2.28 million) in 2019. Based on the production and product value, durian has the highest product value than the other 20 local fruits listed. Banana production (325,447 mt) was higher than pineapple production (314,627 mt) but had a lower product value. Since banana can be easily damaged during its harvesting to its transportation process (Cao et al., 2018), the decrease in production value might be due to the quality losses of postharvest fruits during storage and transportation that eventually lead to reduced prices in the market.

**Table 1:** Production value of major tropical fruits in Malaysia, 2019.

Fruits	Production (mt)	Production Value (RM’000)
Durian	377,251	7,493,882
Banana	325,447	579,295
Pineapple	314,627	621,389
Watermelon	144,147	212,617
Rambutan	55,891	124,489
Papaya	53,681	118,630
Guava	35,962	117,740
Jackfruit	31,281	107,863
Mangosteen	28,764	99,843
Cempedak	27,893	86,096
Duku	24,446	61,058
Dokong	22,913	51,118
Langsat	18,993	40,335
Mango	16,509	39,561
Pomelo	15,133	38,588
Sweet orange	11,006	31,300
Starfruit	8,054	26,415
Dragon fruit	6,879	20,535
Salak	3,443	6,541
Sapodilla	1,828	5,209
Pulasan	966	2,897
Total	1,525,051	9,885,315

Source: Department of Agriculture, Malaysia (2019).

**Figure 2:**
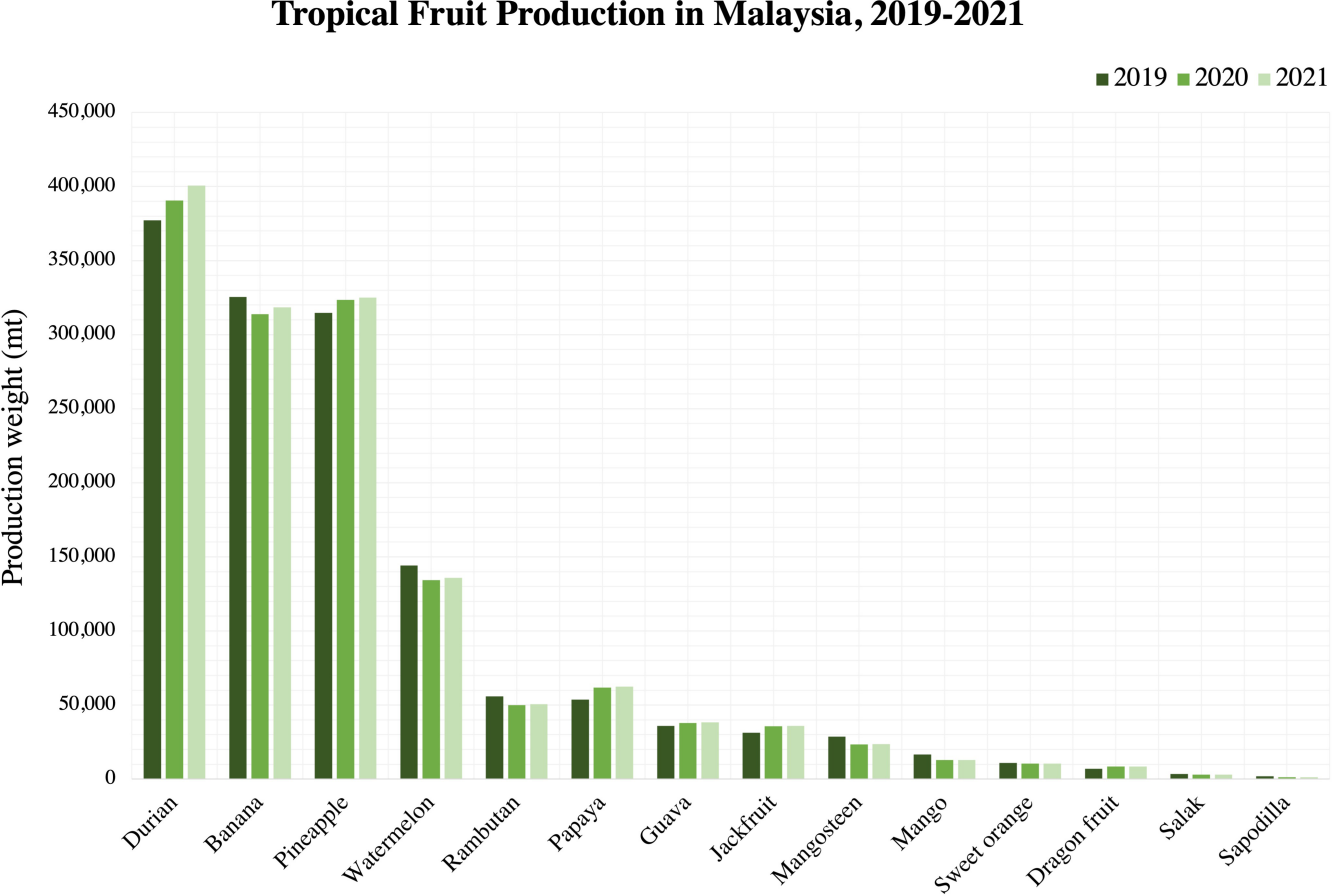
Tropical fruit production in Malaysia, 2019-2021. Source: Department of Agriculture, Malaysia (2021).

From **Figure 2**, the production trend of tropical fruits in Malaysia can be seen to generally increase over the years from year 2019 to year 2021. This indicates an increased demand for tropical fruits where it will be globally exported to different countries. According to Cao et al. (2018), tropical and subtropical fruits are very vulnerable to the surrounding temperature which will make it further susceptible to fungal infections, leading to reduced quality and decay. FAO (2022) reported the decrease of papaya exportation in Malaysia by approximately 4% in year 2022 was partly due to a phytopathogenic bacteria causing the bacterial dieback disease in papaya. Since fluctuations in temperature are inevitable during storage and transportation, there is a need to protect the postharvest tropical fruits to enhance their longevity and consequently, preserve the quality.

Fruits and vegetables are generally considered more perishable than other mass-produced commercial crops like oil palm and rubber. A study of 284 participants found that 93% of consumers consider freshness the top criterion when purchasing and consuming fresh fruits and vegetables (Chamhuri and Batt, 2015). Physical attributes of fruits and vegetables, such as the size, shape, color, and texture, all affected by infections, also contribute to how consumers perceive product quality. This presents a significant challenge for local farmers and exporters to ensure the freshness of crops between getting them from the farm to the consumers. Infections and diseases are also one of the main factors contributing to poor crop yield in Malaysia (Chang, 2021). Due to their higher water content (70-95%), a higher concentration of polysaccharides and significantly increased respiration rate; fruit crops provide a more favorable environment in which microorganisms can thrive. With an average shelf life of 3-5 days, there is a pressing need for the development of highly effective, yet sustainable and safe methods of preserving the freshness of tropical fruits (Wongs-Aree and Noichinda, 2014).

Data and information above indicate the increasing trend in the production of tropical fruits in Malaysia and reveal tropical fruits as important trade products in Southeast Asian countries. In the case of more exotic fruits such as rambutan and durian, the low availability of high-quality fruits as a result of prolonged export times may ultimately drive market prices up, especially in regions where they are not locally cultivated. This then, may affect customer demand, which is inversely proportional to the market price of a product, according to the law of demand. Therefore, the decline in fruit quality during transport and storage necessitate action as the tropical fruit industry play a significant economic role for developing countries in the Southeast Asian region.

## Application of essential oils or plant extracts to protect selected tropical fruits

Essential oils and extracts from plants have been studied in various ways for tropical fruit disease management from cultivated to postharvest processing and storage (**Supplementary Table 2**). The majority of the research done on preharvest disease prevention and treatment is centred around *in vitro* studies. Herein, we reviewed *in vitro* studies utilising a variety of tests. The disc volatilisation method is a popular method of studying the antimicrobial activity of essential oils and extracts (Kloucek et al., 2012). It involves the use of the vapour phase of essential oil of interest through drying on to a piece of material, typically a filter paper. However, to study the effects of essential oils in liquid form, with or without dilution, other techniques such as agar diffusion assays, the poisoned food technique and the broth dilution method can be employed (Balouiri et al., 2016). A small number of studies on the use of these extracts during cultivation *in vivo* were also reviewed, mainly in the form of a protective vapour treatment. Next, plant extracts and essential oils have been assessed to help prevent spoilage and improve the longevity of postharvest fruit products (El Khetabi et al., 2022). The preferred method of utilising essential oils to increase the shelf life of freshly harvested and cut fruits is through the incorporation into edible coating. Various formulations coatings with essential oils and extracts have previously been studied such as nano-emulsions and biopolymer-based coatings. Finally, they have also been studied as potential preservatives in fruit juices, with an additional focus on the potential effects on the sensory attributes like taste and appearance.

### Durian

Durian (*Durio zibethinus*) is an edible fruit belonging to the genus *Durio*. It is grown in the tropics and is characterized by its hard, spiky outer shell. Durian is often exposed to fungal infections, causing various diseases to the crop. *Phytophthora palmivora* is a type of fungus that causes stem canker; *Rhizoctonia solani* is a soil-borne pathogenic fungus that causes leaf blight in durian trees; meanwhile, stem rot in durian trees is caused by *Fusarium solani*. Several studies have been conducted to evaluate the potential antifungal activity of essential oils and plant extracts against these types of fungi *in vitro*. However, to the best of our knowledge, the utilization of essential oils or plant extracts to protect durian *in vivo* has not been studied.

Some essential oils and plant extracts have the potential to be formulated into organic fungicides to prevent stem canker in durian trees by inhibiting the growth of *P. palmivora* as tested *in vitro*. A recent study shows that the vapour of clove and citronella oils can slow down *P. palmivora* growth *in vitro* (Istianto and Emilda, 2021). This is due to the presence of eugenol, a major constituent of volatile clove oil that is believed to possess antifungal properties. Phenolic compounds in clove oil can inhibit the mycelial growth of *P. palmivora* by penetrating the fungal cell membrane and lipids. Hence, the compounds can access the cell’s internal contents and disrupt the protein syntheses in the fungal cell (Ansari et al., 2013). At 10 mg/mL concentration, the n-hexane extract of clove buds and clove oil exhibited 90.0% and 72.7% growth inhibition, respectively, against *P. palmivora*. This study also concluded that despite having the same major antifungal compound, the n-hexane extract of clove buds is more suitable for using organic fungicide than clove oil due to its stronger antifungal activity. The synergistic effect of eugenol and other antifungal compounds in the n-hexane extract might contribute to higher efficacy in fungal growth inhibition (Aulifa et al., 2015). Also, the extract of *Cosmos caudatus*, commonly known as king’s salad, in ethyl acetate recorded only 15.6% germination of *P. palmivora* compared to the control. This might be due to the ability of ethyl acetate to isolate secondary metabolites such as sesquiterpenes, lactones, stigmasterol and lutein from the crude extract that may contribute to the antifungal activity (Mohd Salehan et al., 2013). According to Garcia-Rellán et al. (2016), *P. palmivora* is sensitive to the essential oil extracted from *Satureja cuneifolia*, an aromatic plant used in Turkey to make herbal tea, as it inhibited 77.1% of the fungal growth at 1 mg/mL concentration. The extract from *Hydnocarpus anthelminthicus*, a tree found in the rainforests of Southeast Asia, also showed complete growth inhibition against *P. palmivora* and *R. solani* at a concentration of 10 mg/mL (Jantasorn et al., 2016). The extract’s antifungal properties were mainly attributed to flavonoids, isoflavonoids, phenolics, phenol acids, coumarins and alkaloids.

Apart from preventing stem canker, essential oils and plant extracts can also potentially protect durian trees from leaf blight by inhibiting *R. solani* growth. According to a study carried out by Osman Mohamed Ali et al. (2017), the development of nano emulsions based on a combination of neem and citronella oils is proven to be potential organic fungicides that can control diseases caused by *R. solani*. It was reported that neem and citronella nano emulsions inhibited 40-80% growth of *R. solani* after four days of incubation. Another study reported that more than 80% of *R. solani* growth was inhibited when treated with *Asarum heterotropoides* var. *mandshuricum* essential oil *in vitro* (Dan et al., 2010). The antifungal activity of *Hypericum linarioides* Bosse essential oil against *R. solani* was also evaluated. The acetone and methanol extracts of *H. linarioides* inhibited 43-70% growth of *R. solani*, while pure *H. linarioides* essential oil showed 87.5% growth inhibition at 5 mg/mL (Cakir et al., 2005). In contrast to the previous study by Cakir et al. (2004) that showed α-pinene might contribute to the antifungal properties possessed by *H. linarioides* essential oil, this present study recorded the absence of α-pinene, suggesting the antifungal properties of this essential oil may be contributed by other compounds.

Stem rot and leaf blight caused by *F. solani* and *R. solani* are considered vital diseases in durian cultivation. A study reported that *Piper chaba* Hunter extract containing α-humulene, caryophyllene oxide, viridiflorol, globulol, β-selinene, spathulenol, (E)-nerolidol, linalool and 3-pentanol as antifungal components inhibited 70.3% growth of *R. solani* and 56.6% growth of *F. solani* when tested *in vitro* (Rahman et al., 2011). In another study, the essential oil from *Cuminum cyminum* oil possessed significant antifungal activity against *F. solani* due to pinene, cineole and linalool (Naeini et al., 2010). *Myrcia ovata* Cambessedes essential oil may also be an alternative fungicide to control *F. solani* to prevent stem rot in durian trees. An *in vitro* study showed that *M. ovata* essential oil completely inhibited mycelial growth at a concentration of 30 μL/mL. The authors suggested that this observation may be due to the oil components such as linalool, nerolic acid, geraniol, neral, geranial, (E)-nerolidol, 1,8-cineole and isopulegol (Sampaio et al., 2016). These studies illustrate that essential oils and plant extracts have the potential to be commercialized as botanical fungicides to protect durians from fungal infections. The efficiency of essential oils and plant extracts to inhibit fungal growth *in vitro* suggests that they may be used as an alternative to synthetic fungicides to control the durian trees’ diseases caused by these phytopathogenic fungi.

### Banana

Banana (*Musa paradisiaca*, *Musa acuminaia* or *Musa balbisiana*) is an edible fruit produced by plants of the genus *Musa* and grown in the tropics. The banana fruit is commonly eaten raw and is one of the most economically important tropical fruit crops. However, anthracnose and crown rot are postharvest diseases in banana fruits that reduce their quality. These postharvest diseases are caused by *Colletotrichum* spp. and *Fusarium* spp., respectively. Postharvest decay reduces the quality of banana fruits and is one of the biggest factors of economic losses. Several studies have been carried out *in vivo* and *in vitro* to investigate the efficacy of using essential oils and plant extracts in protecting banana fruits from postharvest diseases.

An *in vitro* study reported that the ethanolic extract of *Eucalyptus camaldulensis* at the concentrations of 0.5 mg/mL and 5 mg/mL inhibited the growth of *C. gloeosporioides* by 50% and 98%, respectively (España et al., 2017). Another *in vitro* study reported that the vapours of essential oil extracted from *Cinnamomum cassia*, commonly known as Chinese cinnamon, at volumes of 5 μL and 6 μL per 90x15 mm plate could completely inhibit the growth of *Lasiodiplodia theobromae* and *Colletotrichum musae* respectively. Holy basil oil also recorded complete inhibition against *L. theobromae* growth at 6 μL per 90x15 mm plate. The study used a modified disc volatilization method where a fixed concentration of tested essential oils (200 mg/mL) were added onto filter paper discs and were let to vaporize in sealed Petri plates containing agar inoculated with *L. theobromae* and *C. musae*. It was proposed that eugenol, cinnamyl acetate, humulene, trans-calamenene and caryophyllene present in both *Cinnamomum cassia* essential oil and holy basil oil, can damage the fungal cell wall and cell membrane, thus altering the membrane potential, which eventually leads to growth inhibition (Kulkarni et al., 2021).

*In vivo* studies need to be done to demonstrate further the potential of using essential oils and plant extracts as organic fungicides against anthracnose. A study showed that the methanolic extract of ginger containing α-curcumene and zingerone as active compounds inhibited more than 80% of *C. musae* growth at a concentration of 5 mg/mL when tested *in vitro*. Banana fruits treated with the extract also recorded a low score of anthracnose severity of 2.2 after five days of storage, compared to the untreated control, which scored 4.8 (Bhutia et al., 2016). Monoterpenes such as citral, L-carvone and citronellal may be used as the active compounds for synthetic fungicides, which completely inhibit *C. musae* conidia germination at concentrations as low as 2 mg/mL, 4 mg/mL, and 2mg/mL, respectively. They are significantly more potent compared to benomyl; a synthetic fungicide commonly used in agriculture that was found to be only able to inhibit 79% of the *C. musae* conidia germination *in vitro*. This shows that these monoterpenes can be formulated into synthetic or chemical fungicides such as benomyl, to enhance their efficacy in killing fungi. Bananas treated with citral showed a 60% reduction of anthracnose lesion diameter compared to the untreated control. It was previously found that citral can negatively affect the tricarboxylic acid cycle, alter mitochondrial morphology, and cause metabolic disorders in pathogenic cells, inhibiting fungal growth and sporulation (Garcia et al., 2008). According to Vilaplana et al. (2018a), bananas treated with 500 μL/L thyme oil showed a 46.4% decay reduction compared to the commercially available fungicide Imazalil, which showed only a 29.4% decay reduction. The authors proposed that the synergistic effect of thymol and carvacrol in thyme oil induced the leakage of the fungal cell membrane, leading to fungal cell tissue deterioration. The fruits also showed better firmness, sensory qualities, and higher weight loss reduction during cold storage than bananas treated with Imazalil. A study reported that incorporating 4 mg/mL cinnamon oil into 100 mg/mL gum arabic can control 80% of anthracnose incidence in postharvest bananas and significantly reduced the weight loss by 89% compared to untreated bananas after 28 days of cold storage. It was also reported that this mixture inhibited 88% of *C. musae* growth when tested *in vitro* (Maqbool et al., 2011). Aloe vera incorporated with garlic oil inhibited 87.7% mycelial growth and 91.2% spore germination when tested *in vitro* against *C. musae*. The mixture was also tested as an antimicrobial coating, which was then found to reduce the incidence and severity of anthracnose by 92.5% and 81%, respectively (Khaliq et al., 2019a).

Crown rot disease is a type of fungal infection that initially occurs at the crown part of bananas and may spread to other parts of the fruit. It is often caused by *Colletotrichum musae* or *Lasiodiplodia theobromae*. Some essential oils and plant extracts have been found to have a similar antifungal activity to commercial fungicides used against crown rot disease. Jahan et al. (2019) reported that the methanol extract of garlic, *A. sativum*, has similar fungicidal activity as chemical fungicides like carbendazim and kanamycin B against crown rot. Spraying emulsions of basil oil on bananas was observed to control anthracnose and crown rot in bananas stored for 21 days. Interestingly, no significant differences were reported compared to benomyl treatment. It also did not affect the physicochemical and sensory properties of treated bananas (Anthony et al., 2003). In another study, eugenol in basil oil controlled crown rot by inhibiting appressorium formation of *C. musae*, which is crucial to initiating an infection (Siriwardana et al., 2017). Moreover, the synergistic effects of *Cymbopogum nardus* oil and basil oil in a liquid medium are more effective in controlling crown rot in bananas than in benomyl treatment. This may be attributed to various antifungal components such as α-pinene, citronellol, citronellal, eugenol and geraniol (Anthony et al., 2004).

Spraying 40 mg/mL cinnamon and 40 mg/mL thyme oil completely controlled crown rot incidence in bananas. The study also found an 87.1% and 78.7% reduction in crown rot incidence when bananas were treated with sweet almond and bitter almond oil, respectively, without altering the organoleptic properties (Abd-Alla et al., 2014). Complete inhibition of crown rot disease in bananas was recorded when treated with a 250 mg/mL concentration of Zimmu leaf extract without altering organoleptic properties. The extract treatment also was found to have better fungicidal activity than the benomyl in reducing crown rot severity (Sangeetha et al., 2013). The cinnamon extract inhibited 25% of crown rot disease in bananas without affecting postharvest quality (Win et al., 2007). An *in vitro* study carried out by Kamsu et al. (2019) found that cinnamon oil inhibited 100% conidial germination of *C. musae*, *Fusarium incarnatum* and *Fusarium verticillioides* at concentrations of 1025, 950 and 9088 μL/L respectively. *C. musae*, *F. incarnatum* and *F. verticillioides* conidial germination were also completely inhibited by lemongrass oil at 200, 185 and 275 μL/L, respectively. The germination inhibition may be due to the terpenes that act as antifungal compounds, disrupting fungal germination in essential oils. The previous results were validated when Ranasinghe et al. (2002) also found that cinnamon and clove oils possess fungicidal properties against *C. musae*, *L. theobromae* and *F. proliferatum* when tested *in vitro*.

These studies suggested that essential oils and plant extracts have the potential to be organic fungicides in controlling anthracnose and crown rot disease in postharvest bananas. They also can extend the shelf life of postharvest bananas by improving the physicochemical properties without interfering with the organoleptic properties or sensory qualities.

### Pineapple

Pineapple (*Ananas comosus*) is a tropical plant from the family *Bromeliaceae*. It has spiky leaves on top and tough leathery skin. Fusariosis is a type of fungal infection that commonly affects pineapple plants. *Fusarium* spp. is the common fungus responsible for fusariosis in pineapples. Fresh-cut pineapples and pineapple juices are susceptible to mould and yeast contamination that causes spoilage. Several studies have been conducted to evaluate the uses of essential oils and plant extracts as natural fungicides to protect pineapples from fusariosis and as alternative preservative methods to control postharvest spoilage.

Essential oils and plant extracts can be used to control fusariosis in pineapples. A recent study reported that monoterpenes such as citral, L-carvone, and citronellal could be a potential alternative for synthetic fungicide as they completely inhibited *Fusarium subglutinans* f.sp *ananas* germination at a concentration as low as 4 mg/mL, 8 mg/mL and 6 mg/mL respectively compared to commercially available chemical fungicide, benomyl that has lower conidia germination inhibition of 42% when tested *in vitro* (Garcia et al., 2008). It shows that citral has a very high antifungal characteristic. It was proposed that citral enter the cell by inducing malondialdehyde, reducing cell membrane elasticity. Then it alters the citric acid cycle and mitochondria morphology which subsequently inhibits fungal growth and sporulation (Luo et al., 2004). A study reported that thyme oil inhibited 100% of *Fusarium verticillioides* mycelial growth at a concentration as low as 250 μL/L when tested *in vitro*. As for *in vivo* study, postharvest pineapples treated with 1000 μL/L thyme oil showed 50.1% disease reduction in 7 days of storage without affecting the sensory quality, higher than fruit treated with chemical fungicide, prochloraz, which showed 32.7% disease reduction (Vilaplana et al., 2018b).

Cutting fruits increases their metabolic activity, thus reducing their shelf life. It also increases the susceptibility to microbial contamination and lowers the quality of the fruit. The development of edible coatings incorporated with essential oils or plant extracts can act as a barrier that protects fresh-cut pineapples from microbial contamination, prolongs shelf life and maintains their quality. Some major essential oil compounds are difficult to incorporate into food due to their lipophilic nature. To overcome this challenge, lipophilic compounds must be emulsified into nano emulsions to be easily incorporated into edible coatings for fruit protection. A study conducted by Prakash et al. (2020) reported that edible alginate coatings incorporated with 0.5 mL/100mL and 1 mL/100mL of citral nano emulsions inhibited *Salmonella typhimurium* and *Listeria monocytogenes* total plate count growth by 4.68 log CFU/g and 2.77 log CFU/g respectively compared to control which recorded higher than 7 log CFU/g. It was also observed that the colour and appearance of the coated cut pineapples were enhanced, which may be contributed by citral inhibiting polyphenol oxidase activity in the coated fruits. This shows that the major compound citral found in various plant essential oils, such as lemongrass essential oil has antimicrobial activity. Another study showed that incorporating 3 mg/mL lemongrass essential oil into alginate coating reduced the weight loss in coated fresh-cut pineapples during storage. It was proposed that the lipophilic nature of essential oils can reduce respiration rate, reducing the weight loss in coated fruits (Azarakhsh et al., 2014). de Araujo et al. (2021) reported that the shelf life of fresh-cut pineapples coated with chitosan incorporated with black pepper (*Piper nigrum*) and Brazilian pepper (*Schinus terebenthifolia*) essential oil was improved by 45 days and recorded 98.4% efficiency in reducing microbial counts such as *E. coli* and *S. aureus.*

Pineapple juice is susceptible to spoilage caused by mould or yeast contamination. A study reported that sodium benzoate and citrus extract could be used during fruit juice homogenization as antimicrobial preservatives to reduce spores of *Fusarium oxysporum*. Curiously, *F. oxysporum* is a type of fungus that is typically resistant to homogenization. The citrus extract reduced spore counts to 1.14 CFU/mL at a concentration of 1.5 mg/mL and completely removed the spores at a concentration of 3 mg/mL compared to the control, which recorded 6 CFU/mL spore counts. It was proposed that terpenes in the citrus extract can increase peroxide concentration, causing the breakdown of the cell wall and destroying the fungus’s vegetative reproduction (Bevilacqua et al., 2012). An investigation by da Cruz Almeida et al. (2018) reported that essential oils from spearmint (*Mentha spicata* L.) and Bowles mint (*Mentha × villosa* Huds) can be used in the preservation of pineapple juices against spoilage yeasts. A reduction of *Pichia anomala* and *Saccharomyces cerevisiae* was observed when pineapple juices were treated with 3.75 μL/mL of *M. spicata* essential oil (MSEO) after 48 h of exposure. A reduction in *S. cerevisiae* was observed when treated with 15 μL/mL of *M. x villosa* essential oil (MVEO). This might be due to the antifungal components found in MSEO and MVEO, such as carvone and piperitone oxide. It was proposed that carvone inhibits the proton pump, and the biosynthesis pathway of ergosterol in fungal cells eventually disturbs the cell integrity (Samber et al., 2015). At the same time, piperitone oxide could disrupt the cell membrane hence altering the metabolic activity of the fungus (Ait-Ouazzou et al., 2012; Guerra et al., 2015). These studies present that the antifungal activity of essential oils and plant extracts can be utilized to protect pineapple plants from fusariosis, improve the quality of processed pineapples and control the spoilage in pineapple juices.

### Watermelon

Watermelon (*Citrullus lanatus*) is a flowering fruit from the family *Cucurbitaceae*. The crop is cultivated globally but thrives in tropical climates such as that near the equator. It is commonly characterized by a large round fruit protected by a hard outer skin painted with green stripes. Watermelons are exposed to pests and viral infections, while processed watermelons tend to get spoiled due to higher enzymatic reactions. A few studies that have been carried out over the past few years found that essential oils and plant extracts can be utilized to protect fresh and processed watermelons from harmful pests and pathogenic microbes.

*Bactrocera cucurbitae*, commonly known as the melon fly, is a watermelon pest that causes significant losses to farmers. Melon flies lay their eggs on the watermelon fruit and once hatched, maggots will feed on the fruit, damaging it internally and causing it to quickly rot. Instead of using chemical insecticides to control the infection of *B. cucurbitae*, some plant extracts can be utilized as potential biopesticides that could target the pests at their earlier stage of development. A study was carried out to investigate the larvicidal and pupicidal activities of neem (*Azadirachta indica*), Chinese chaste tree (*Vitex negundo*) and water pepper (*Persicaria hydropiper*) methanolic extracts against *B. cucurbitae in vitro*. It was shown that exposure to *A. indica*, *V. negundo* and *P. hydropiper* recorded high mortality degrees of *B. cucurbitae* (LD_50_ 1.161 mg/cm^2^, 2.213 mg/cm^2^ and 0.853 mg/cm^2^ respectively) in the larvicidal test. *A. indica* and *P. hydropiper* extracts also recorded significantly high pupicidal activities against *B. cucurbitae* (LD_50_ 0.26 mg/cm^2^ and LD_50_ 8.70 mg/cm^2^ respectively) (Hossain and Khalequzzaman, 2018).

Apart from the damage caused by *B. cucurbitae*, watermelon crops are also susceptible to viral infections such as the watermelon mosaic virus which affects their overall physical features such as yellow spots on leaves, stunted growth, severe discolouration and in extreme cases, necrosis (Xu et al., 2004; Desbiez and Lecoq, 2021). Some plant extracts have been discovered to effectively prevent viral diseases in watermelons by activating defence mechanisms in the treated fruits. A study demonstrated that seed treatment of watermelons followed by six foliar sprays using *Boerhaavia diffusa* root, *Clerodendrum aculeatum* leaf, *Azadirachta indica* leaf, and *Terminalia arjuna* bark extracts recorded 54.2%, 45.6%, 52.0% and 34.8% viral disease reduction, respectively. Increments in vine length, fruit diameter and weight in watermelons treated with these extracts were also observed. The authors proposed that phytoproteins present in the extracts induced a viral resistant mechanism in treated plants by stimulating the production of a viral inhibiting agent (VIA) in the host cells. However, the details of which remain to be fully elucidated. It was also reported that *B. diffusa* may be able to alter the morphology of plant cells to inhibit viral multiplication in host cells (Sharma et al., 2017).

Since fresh-cut and watermelon juices are susceptible to yeasts and mould growth, cinnamaldehyde, mostly found in the essential oil of cinnamon bark, can be used as an antimicrobial agent and employed as an edible coating and preservative. Trans-cinnamaldehyde incorporated into alginate-based coating can act as an antimicrobial compound in multi-layered edible coating to protect fresh-cut watermelons. It was shown that coated fresh-cut watermelons have lower yeasts and mould growth than uncoated fresh-cut watermelons. The coatings also act as a barrier to prevent the respiration rate of the fruit, hence delaying the softening of fresh-cut watermelons and reducing weight loss (Sipahi et al., 2013). Another study also proved the antimicrobial effect of trans-cinnamaldehyde when used as a preservative in watermelon juices, where the solubility was enhanced by nano-emulsification. The study showed that 8 mg/mL trans-cinnamaldehyde inhibited *Salmonella typhimurium* and *Staphylococcus aureus* growth in watermelon juices and extended the shelf life (Jo et al., 2015). These studies show that essential oils and plant extracts can be used as alternative synthetic pesticides and further developed in food preservation techniques.

### Papaya

Papaya (*Carica papaya*) is a tropical fruit from the family *Caricaceae*. It has a sweet taste and juicy flesh, turning orange when ripe. Postharvest papaya is commonly infected by the *Colletotrichum* spp., the fungus which causes anthracnose; meanwhile fresh-cut papayas are prone to mould and yeast contamination. The use of essential oils incorporated into edible coatings has been studied to avoid quality losses of postharvest and fresh-cut papayas.

Coating papayas with *Aloe vera* (AV) can lower the respiration rate of the fruit which subsequently slows down the metabolic process. This helps to delay ripening and increase the shelf life during storage. A study reported that postharvest papayas coated with 50 mL/100 mL of AV gel diluted in distilled water recorded a 2.05% weight loss and 52.29 N of firmness compared to uncoated papayas with a significantly higher 13.2% weight loss and lower firmness of 12.7 N during 15 days of storage. No disease incidence is reported for papayas coated with 50 mL/100 mL AV gel diluted in distilled water after 15 days of storage at a temperature of 28 ± 2°C and 68–70% relative humidity (Mendy et al., 2019). However, a slight increase of relative humidity to a range between 82 to 84%, and decrease of room temperature to 25°C led to 27% disease incidence despite being coated with 100% AV (Brishti et al., 2013). This suggests that although effective, the antimicrobial property of AV gel may be sensitive to fluctuations in room humidity and temperature. Water is generally lost from the papaya fruit through its peel. AV gel coating was found to act as a barrier for water loss, which also contributes to the reduction in fruit weight loss. It was proposed that AV can extend the shelf life of stored fruits by altering their internal environment (Serrano et al., 2005; Valverde et al., 2005). AV gel coating reduces the oxygen availability for oxygen degradation, allowing carotenoid retention.

Another study showed that 20 mg/mL ginger oil incorporated into 100 mg/mL gum arabic used as an edible antimicrobial coating for postharvest papayas recorded lower anthracnose incidence (21%) compared to control (100%) during 28 days of storage. This observation was accompanied by the amelioration of the quality of postharvest papayas without any significant effect on the sensory properties. Using a disease severity scoring scale of 0 to 5, the fungicidal activity of ginger oil was demonstrated when it scored 2.2, less than half compared to the control which reached a maximum score of 5 *in vivo*. This is believed to be due to the antifungal compounds such as α-pinene, 1,8-cineole and borneol present in ginger oil (Ali et al., 2016). These antifungal compounds can lower pathogenic infection by blocking lenticels and cuticles, reducing the respiration and ripening rate of fruits (Martínez-Romero et al., 2006). Limited oxygen availability also reduced the enzyme activity responsible for fruit softening and maintaining fruit firmness (Salunkhe et al., 1991). Postharvest papayas with mesquite-gum based edible coating incorporated with 0.1% (w/w) thyme oil and 0.05% (w/w) Mexican lime essential oil recorded 100% reduction of *C. gloeosporioides* and *Rhizopus stolonifer* infection (Bosquez-Molina et al., 2010). Comparing these two essential oils, Mexican lime oil exhibits higher fungicidal activity than thyme oil, especially when utilized at high concentrations. However, a concentration too high may be poisonous to the fruit which can consequently alter the fruits’ tissue ability to inhibit microbial growth. It is then vital to identify the optimal concentration with maximum antifungal properties yet tolerable to the fruits.

Treating postharvest papayas using carboxymethyl cellulose associated with *Lippia sidoides* essential oil delayed rotting and recorded the lowest minimum inhibitory concentration (0.0753 mg/mL) against *C. gloeosporioides* (Zillo et al., 2018). This is due to the presence of thymol and carvacrol in the essential oil that can modify the fungal cell wall and cell membrane to the point of disrupting the essential growth process of the fungus. Maqbool et al. (2011), incorporated of 4 mg/mL cinnamon oil into 100 mg/mL gum Arabic, which controlled anthracnose incidence by up to 71% in postharvest papayas and significantly reduced 81% of the fruit weight loss compared to untreated papayas. Apart from that, it also inhibited 85% of *C. gloeosporioides* growth when tested *in vitro*. These effects are ascribed to the presence of cinnamaldehyde, which limits microbial growth by disrupting the electron transport chain and reacting with nitrogen-containing compounds (Gupta et al., 2008).

Apart from protecting postharvest papayas, essential oils are also useful in reducing mould and yeast contamination in fresh-cut papayas. Fresh-cut papayas coated with cassava starch-based edible coating and 10 mg/mL lemongrass essential oil effectively suppressed yeasts and mould growth by up to 1.48 log CFU/g. They recorded greater weight preservation than uncoated fresh-cut papayas (Praseptiangga et al., 2017). Another study showed that encapsulated trans-cinnamaldehyde could act as an antimicrobial compound when incorporated into multi-layered edible coating without altering the flavour of fresh-cut papayas (Brasil et al., 2012). Moreover, when fresh-cut papayas were coated with 10 mg/mL psyllium gum and sunflower oil, 5 log CFU/g of mould and yeast count was recorded. Contrarily, more than 10 log CFU/g was recorded for the uncoated counterpart. In addition, the hydrophobic property of sunflower oil is can act as a barrier to water vapour loss, leading to reduced weight loss in the coated fruits (Yousuf and Srivastava, 2015). However, it is important to note that the effectiveness of essential oils in protecting fresh-cut papayas only applies to specific time points. Based on these studies, it is shown that essential oils can be utilized to improve the quality in postharvest and fresh-cut papayas upon storage, mainly through incorporating them into edible external coatings.

### Guava

Guava (*Psidium guajava*) is a tropical fruit from the family *Myrtaceae*. It is a small-sized fruit with a crunchy texture with high contents of vitamin C. Guavas are exposed to fruit pests such as *Bactrocera cucurbitae*, leading to fruit rot and spoilage. Yeasts such as *Pichia anomala* and *Saccharomyces cerevisiae* affect the quality of guava juices. Therefore, essential oils and plant extracts’ effectiveness in protecting guava from pests, extending the shelf life of postharvest guavas, and reducing spoilage in guava juices were studied.

Incorporating essential oils and plant extracts into edible coating can improve the quality and extend the shelf life of postharvest guavas. Formulation of 10 mg/mL pomegranate peel extract in chitosan coating reduced the transpiration rate in coated guavas due to its lipophilic properties. The low transpiration rate maintains the concentration of internal compounds, resulting in only 29% of ascorbic acid, 8% of total phenol and 12% total flavonoid being lost, thereby delaying of the ripening process during storage (Nair et al., 2018). It was also recorded that postharvest guavas coated with aloe vera gel maintained total flavonoid contents, total antioxidant capacity and sensory properties after 12 days of storage at 27 – 29°C (Kumar et al., 2017). Treating guavas with 2.5 mL/100 mL Tulsi extract incorporated into Arabic gum and sodium caseinate inhibited mould growth during seven days of storage at 28°C (Murmu and Mishra, 2017). Another study reported that 2% cinnamon oil and 20 mg/mL lemongrass oil incorporated into 50 mg/mL Arabic gum and 10 mg/mL sodium caseinate extended guava shelf life up to 40 days. It was proposed that geraniol in lemongrass can slow down polyphenol oxidase (PPO) activity by forming hydrogen bonds with active enzymes which reduce browning in treated guavas (Murmu and Mishra, 2018).

*Aloe vera* can be used as an edible antimicrobial coating to protect fresh-cut guavas. Lower weight loss and microbial count were reported on fresh-cut guavas coated with aloe vera than on uncoated fresh-cut guavas. This is due to the antimicrobial compounds such as pyrocatechol, cinnamic acid and p-coumaric acid in aloe vera. The coating can also attract and hold water, preventing water loss and weight loss of guavas (Nasution et al., 2015). In addition to the improvement in the quality of pineapple juice post-storage described above, da Cruz Almeida et al. (2018) reported that the essential oils from spearmint (*Mentha spicata L.*) and *Mentha × villosa Huds* are also able to preserve guava juice against spoilage yeasts in the same manner. This is due to the fact that these different fruit juices are susceptible to infection by a common pathogen, namely *Saccharomyces cerevisiae*.

Based on these studies, it is believed that essential oils and plant extracts are able to prevent the degradation of phytochemicals leading to extended shelf life of postharvest guavas and prevent contamination in the processed fruit juice. The observations also further illustrate the flexibility of essential oil in preventing a range of unrelated crops from postharvest spoilage. However, pre-cautions need to be taken into account during formulation process, especially when incorporating essential oils into secondary products as high concentrations of essential oil or plant extract can be unfavourable to the sensory attributes of the fruit juice.

### Mangosteen

Mangosteen (*Garcinia mangostana*) is a small tropical fruit from the family *Clusiaceae*. It has a purple rind that is both thick and hard to protect a slightly sweet and sour flesh. Postharvest losses of mangosteen can be caused by fungal infections such as *Glomerella cingulata* or gradual fruit ripening and decaying. Several studies have been carried out to investigate the effect of essential oils on mangosteen *in vitro* and *in vivo*.

*In vitro* studies carried out by Permana et al. (2021) showed that emulsions incorporating virgin coconut oil and cinnamaldehyde can impede the growth rate of *Glomerella cingulata* and can potentially be used as an edible coating for mangosteen. In addition, extracts from various plants such as clove buds, pepper, cinnamon, turmeric, ginger, oregano and thyme may also be potentially used in organic fungicides due to their content of eugenol. Due to its poor stability, Velho et al. (2019) studied the nanoencapsulation of eugenol and the synthetic fungicide, mancozeb, and the consequent antifungal activity against *G. cingulata*. It was found that this mixed formulation had increased antifungal efficacy compared to the free forms of eugenol and mancozeb. The toxicity of the resulting formula was tested, and the authors found that it was safe for plant cells and relatively non-toxic in the soil (da Silva Gündel et al., 2019). Mangosteen treated with 2 mL/L of citronella oil also recorded 20% lower scarring symptoms and a lower ant attack percentage compared to untreated mangosteen. Citronella oil can cause death to ants by damaging its integument. Not only that, the presence of odorous compounds such as citronellal, citronellol and geraniol naturally carry the ability to repel insects (Istianto and Emilda, 2021).

According to Owolabi et al. (2021b), postharvest mangosteen treated with peppermint oil and lime oil formulated with a ratio of 1:3 led to fewer fungal infections. They are also observed to ripen slower which can contribute to prolonged shelf life. Another study also noted that tapioca starch incorporated with peppermint oil and lime oil could be applied on rubberwood boxes to preserve postharvest mangosteen during transportation (Owolabi et al., 2021a). Limonene, γ-terpinene, terpinolene, eucalyptol, menthone, and menthol are predicted to be the major components of the mixture that contributed to the remarkable antifungal activity. The process of postharvest ripening can also be influenced by the concentration of ethylene. Previously, it was found that essential oils can suppress the 1-aminocyclopropane-1-carboxylic acid synthase oxidase (ACO) transcription gene that is responsible for ethylene production (Owolabi et al., 2021b). This genetic alteration is one of the many notable modes of action in which essential oils can be utilised to improve the longevity of perishable crops. Hence, it is proven that essential oils can be used as natural pesticides and fungicides to maintain the quality of mangosteen.

### Mango

Mango (*Mangifera indica*) is a sweet tropical fruit belonging to the family *Anacardiaceae*. It varies greatly in shape, colour and taste. The flesh is typically sweet when ripe. In some Asian regions, the fruit is enjoyed when not fully ripened, where it carries a sharp sour taste. *Colletotrichum* sp. is a common anthracnose-causing agent in many fruit crops, including mangoes. Several studies have elaborated on applying essential oils as edible coating to protect and improve the quality of postharvest mangoes. Antifungal properties and the ability of essential oils and plant extracts incorporated into edible coatings to control respiration rate and act as a barrier to water vapour have also been studied in the management of diseases in postharvest mangoes.

Coatings formulated with essential oils and plant extracts were found to reduce diseases in coated mangoes. A test carried out by de Oliveira et al. (2017) evaluated the antifungal effect of *Mentha piperita* essential oil (MPEO) on *Colletotrichum asianum*, *Colletotrichum dianesei*, *Colletotrichum fructicola*, *Colletotrichum tropicale* and *Colletotrichum karstii*. The synergistic effect of the chitosan coating (5 or 7.5 mg/mL) and *M. piperita* essential oil (MPEO) (0.3, 0.6 or 1.25 μL/mL) inhibited 100% of all *Colletotrichum* sp. growth tested on mango fruits. A large portion of MPEO is made up of monoterpenes, such as menthol and isomenthone, which can disrupt the cellular metabolism of fungal cells (dos Santos et al., 2012). The authors proposed that chitosan may be able to alter the fungal cell membrane permeability, allowing antifungal compounds present in MPEO to act on the fungal cell. Mycelial growth percentages of *Colletotrichum* sp. in the range of from 13.5-85.2% were inhibited by 0.3-2.5 μL/mL of MPEO respectively, *in vitro*. This indicates that the antifungal activity of MPEO is concentration dependent, at least up to a concentration of 2.5 µL/mL. Interestingly, mangoes coated with 5 mg/mL and 0.6 μL/mL of chitosan/MPEO recorded lower anthracnose lesion severity than the synthetic fungicide, difenoconazole. Next, ginger oil was investigated as an antimicrobial additive when it was incorporated into a hydroxypropyl methylcellulose coating. When tested for *C. gloeosporioides* growth, the coating showed 42.6% inhibition and consequently, a 38% anthracnose reduction compared to untreated controls after being stored at 25°C for five days. Weight and firmness were also better preserved compared to uncoated mangoes (Klangmuang and Sothornvit, 2018). According to Zhou et al. (2021), 80 mg/mL galangal essential oil incorporated into carboxymethyl chitosan and pullulan coating also prolonged the shelf life of coated mango up to 9 days. After 15 days of storage, mangoes treated with the carboxymethyl chitosan/pullulan coating incorporated with 80 mg/mL galangal essential oil recorded lower weight loss (8.7%) and greater firmness (3.82N) than uncoated mango.

These results were able to indicate the uses of essential oils in the disease control in mangoes at the postharvest stage. Further studies can be done to evaluate the potential of utilising these natural extracts at different points of the mango supply chain. It may be of interest to fully understand how essential oils can affect the sensory properties of mangoes preharvest due to the fact that mangoes are highly variable in taste.

### Sweet orange

Sweet orange (*Citrus X sinensis*) is a hybrid fruit resulting from the cross cultivation of mandarin orange and pomelo. The overall appearance is similar to the typical orange, but sweet oranges are comparably smaller. *Penicillium digitatum* is a type of fungus that causes green mould in postharvest oranges while *Penicillium italicum* causes blue mould. Some fungi such as *Issatchenkia orientalis*, *Meyerozyma caribbica* and *Meyerozyma guilliermondii* are responsible for spoilage in processed orange juice. Several studies evaluated antifungal activity against these postharvest pathogenic fungi by incorporating essential oils or plant extracts into edible coating and preservation methods.

Essential oils incorporated into edible coatings can reduce the disease severity of coated oranges. 0.361 g/mL of pomegranate peel extract incorporated into chitosan and locust bean gum also recorded a green mould incidence reduction (95% and 75%, respectively) when used as coatings for sweet orange compared to uncoated control. The high phenol content in the pomegranate peel extract is believed to be driving the reduction in green mould incidence (Kharchoufi et al., 2018). Another study reported the incorporation of tea tree oil into chitosan coating reduced 50% of *P. italicum* growth on artificially inoculated oranges compared to uncoated fruits. The authors also incorporated bergamot oil into the coating, which added the effects of preserving the weight and firmness of the fruit (Cháfer et al., 2012).

Adeogun et al. (2016) reported that ethanolic extract of a herbaceous plant in Africa, commonly known as the miracle berries or *Thaumatococcus daniellii*, could potentially be used to protect sweet orange juice against spoilage yeasts *in vitro*. It was reported that the minimum inhibitory concentration for the ethanolic extract of *T. danielli* against *I. orientalis*, *M. caribbica* and M*. guilliermondii* are as low as 0.1, 0.5 and 0.1, respectively. Ethanol can be a useful solvent in extracting plants’ antimicrobial compounds of plants to be used as antimicrobial preservatives in fruit juices. As ethanol is acidic, it can make a medium more acidic by donating a hydrogen ion in the aqueous state. Microorganisms present in the medium tend to take up the hydrogen ion, leading to increased concentration of hydrogen ions inside the microbial cells and subsequently, death. Hence, the potency of essential oils or plant extracts to replace chemical fungicides in agriculture and food preservation is proven.

### Other tropical fruits: Rambutan, jackfruit, dragon fruit, salak and sapodilla

Rambutan, jackfruit, dragon fruit, salak and sapodilla are fruits that are mostly cultivated in tropical climates, typically within the Southeast Asian region. They, like many other fruit crops, are highly prone to fungal infections. Several studies have been carried out to investigate the efficiency of essential oils and plant extracts against phytopathogenic fungi in the form of edible coating and organic fungicides.

Rambutan (*Nepphelium lappaceum*) has a juicy white flesh protected by typically a red or yellow hairy outer skin. *Oidium nephelii*, is a pathogen of the rambutan fruit, causing powdery mildew at the preharvest stage of cultivation. In addition, rambutan is also susceptible to other more common fungal infections that cause postharvest diseases, such as *Colletotrichum gloeosporioides*, *Gliocephalotrichum microchlamydosporum, and Botryodiplodia theobromae* leading to anthracnose, brown spot and stem end rot, respectively.

The extracts of wood vinegar and *Curcuma longa* (turmeric) were previously studied as alternatives to chemical fungicides in controlling powdery mildew. An *in vitro* study showed that *O. nephelii* germination was completely inhibited when treated with 0.5 µL/mL of wood vinegar extract and 0.5 g/mL extract of *Curcuma longa*. The fungicidal effects of both extracts were further investigated through *in vivo* studies when treatment with *Curcuma longa* and wood vinegar extract recorded 13.8% and 9.3% of infection severity, respectively. This pales in comparison to the untreated control which recorded an infection severity of 61.1% (Preecha et al., 2017). Rambutan fruits treated with clove oil for 13 days exhibited complete inhibition of powdery mildew infection compared to the untreated control *in vitro*, where the latter grew by 40-fold in colony size (Istianto and Emilda, 2021). The reason for this inhibition is believed to result from the alteration of fungal cell surface and structure by clove oil which inhibits the development of the fungus. However, the study also found that at high concentrations (4 mg/mL), rambutan fruit damage was observed, suggesting a potential phytotoxic effect of the clove oil. In postharvest rambutan, cinnamaldehyde was reported to be effective against common pathogens such as *C. gloeosporioides*, *G. microchlamydosporum* and *B. theobromae*. Complete inhibition of mycelial growth and spore germination of all three fungi were recorded when treated with cinnamaldehyde at 0.03 mg/mL and 0.05 mg/mL *in vitro*; meanwhile, an *in vivo* study recorded reduced disease severity in rambutan when treated with the same concentration (1.5 cm lesion diameter) compared to untreated control (4 to 4.5 lesion diameter) (Sivakumar et al., 2002).

Jackfruit (*Artocarpus heterophyllus*) is a tropical fruit characterized by bumpy outer skin, stringy core and multiple seeds with yellow coloured flesh. The fruit has been gaining much attention as a meat replacement in vegetarian and vegan communities, making it an economically important export. They are, however, extremely prone to rotting and spoilage, hence the immediate need for effective control measures during transport and storage. Postharvest rot in jackfruits caused by *Penicillium notatum* may be prevented by using basil (*Ocimum basilicum*), and *Vetiveria zizanioides* essential oils. A study carried out by Atif et al. (2020) reported that vapour treatment using the mixture of *O. basilicum* and *V. zizanioides* essential oils at 25 µL concentration reduced *P. notatum* colony area to 4.2 ± 1 cm^2^. It completely suppressed the spore germination after seven days when tested *in vitro*. *P. notatum* growth was also reduced when postharvest jackfruit was treated using the essential oil vapour. The presence of L-carvone and phenolic compounds in the essential oils may play significant roles in the mycelial growth inhibition of *P. notatum*.

Dragon fruit (*Hyelocereus megalanthus*) is a sweet-tasting fruit with small edible seeds. The fruit possesses a soft, scaly outer skin of various colours ranging from red, and purple to yellow. *Alternaria alternata* is a common fungus that causes postharvest disease in dragon fruit; meanwhile, anthracnose in dragon fruit is caused by *C. gloeosporioides* and *Colletotrichum fructicola*. Cinnamon (*Cinnamomum zeylanicum*) and clove (*Eugenia caryophyllus*) essential oils visually inhibited *A. alternata* growth *in vitro* with concentrations as low as 0.25 mg/mL and 0.5 mg/mL, respectively. Meanwhile, dragon fruit treated with 500 μg/mL *E. caryophyllus* essential oil recorded a 31% reduction of mycelial growth compared to untreated fruit *in vivo*. A separate study against *C. gloeosporioides* showed that 10.0 mg/mL of ginger extract in ethanol inhibited 88.5% of mycelial growth and 87.5% of conidial germination. Gingerol, the compound responsible for the distinctive ginger taste, may be responsible for the antifungal activity of ginger oil. While effective at controlling the growth of *C. gloeosporioides*, a higher concentration of ginger oil is needed when compared to the commercial fungicide, mancozeb, which recorded an inhibition of 80.7% at a much lower dosage of 2 mg/mL. Dragon fruit treated with 10 mg/mL of turmeric extract and “dukung anak” extract controlled anthracnose incidence postharvest during 28 days of storage compared to control due to the action of curcumin and alkaloids present in the extracts. Gingerol, curcumin and alkaloids can disrupt the fungal cell wall, causing the leakage of electrolytes which leads to the death of the fungus. However, at higher concentrations, “dukung anak” extract and turmeric extract can exhibit phytotoxicity by damaging the fruit’s cell tissue and allowing phytopathogenic organisms to attack the damaged fruit (Bordoh et al., 2020). Next, another study reported that 400 μL/L of carvacrol essential oil completely inhibited *C. fructicola in vitro* when treated fruits recorded a lower lesion (7.6 mm) than untreated fruits (27.33 mm). It was also reported that treatment with carvacrol increased the concentration of malondialdehyde, a marker for oxidative stress. The authors proposed that carvacrol can increase the production of reactive oxygen species, leading to lipid peroxidation and increased membrane permeability in *C. fructicola*. It also can modify cell permeability, leading to the unintentional exchange of intracellular components and eventually causing death in fungal cells (Pei et al., 2020).

Salak (*Salacca zalacca*) is a fruit with tough, scaly, and prickly reddish-brown outer skin. The white flesh tastes sweet when fully ripened but tastes sour if consumed unripe. A recent study showed that application of 0.08% (w/w) orange oil vapour in a closed air system completely inhibited *Marasmius palmivorus* and *Thieviolopsis* sp. growth on salacca fruit due to the presence of limonene and subsequently extended the shelf life up to 28 days. Due to its ability to pass through the fungal cell membrane, limonene can disrupt protein synthesis in the fungus and subsequently inhibit fungal sporulation and germination. (Phothisuwan et al., 2021).

Sapodilla (*Manilkara zapota*) is a brown and round or oblong-shaped fruit with a sweet taste with gritty textured flesh, almost like that of a kiwi. Plant extracts can be incorporated into coatings to preserve postharvest sapodilla. Khaliq et al. (2019b) reported that the formulation of 100% aloe vera and 10 mg/mL *Fagonia indica* extract could be applied as a coating to extend the shelf life and preserve sapodilla during storage. Treated sapodilla recorded 9.3% weight loss, higher firmness level (6.67N) and lower decay incidence (4.3%) compared to untreated sapodilla (22.1%, 3.65N and 34.7% respectively). Rambutan, jackfruit, dragon fruit, salak and sapodilla are considered rather exotic fruits, even in regions where they are cultivated. Therefore, studies looking into the use of natural preservatives for the disease management of sapodilla crops remain scarce. With that being said, the limited studies are convincing to demonstrate the versatility of using essential oils and plant extracts as natural disease-controlling mechanisms in a range of tropical fruit crops.

## Conclusion and future directions

Essential oils and plant extracts have shown the potential to protect and enhance the quality of pre and postharvest fruits owing to their antimicrobial properties. Some of the essential oils and plant extracts can be used to be formulated as organic fungicides to control diseases in preharvest fruits. As for postharvest fruits, essential oils and plant extracts can be developed into edible coatings incorporated with antimicrobial agents for protection during storage and transportation. Coatings can also be designed for shorter-term storage to prevent rapid spoilage of processed products such as fresh-cut fruits. As essential oils are generally lipophilic while plant extracts are typically extracted in organic solvents, the respiration rate of coated postharvest fruits may be significantly reduced due to the limited exchange of gases. This, in turn, results in the extension of their shelf life, allowing exporters to limit the use of synthetic preservatives. Essential oils and plant extracts may be utilized as organic preservatives for fruit juices, apart from protecting preharvest, postharvest and processed fruits. Hydrophilic compounds with antifungal activities present in essential oils and plant extracts can distribute evenly in fruit juices due to the high water content, increasing their chances of interaction with microorganisms.

The effectiveness of essential oil or plant extract-based edible coating is still, however, dependent on the humidity and temperature of the storage environment; thus, more studies are needed to design more reliable and robust protective coatings effectively. More studies are also warranted to understand the formulation of essential oils and plant extracts and organic pesticides without diminishing their antimicrobial properties. Experiments investigating the antimicrobial properties of these oils and extracts are also concentrated on the direct implications to the pathogens; thus, the effects on the actual fruit need to be thoroughly understood through *in vivo* studies. Finally, although generally considered less toxic than chemical pesticides, the safety of essential oils and plant extracts in formulated coatings must be confirmed before they can be applied in the fruit industry.

## Author contributions

Data curation, original draft preparation, writing revisions, editing, visualisation, NI, MM. Resources and review, SM, XS, AA, SC’N. Supervision and project administration, BG, ST, NI, KC. All authors have read and agreed to the published version of the manuscript.

## Funding

This work was supported by the collaborative project between Universiti Putra Malaysia and MyPower Biotech Sdn. Bhd. (Malaysia) (Project vote number/ project code: 6300261-12038).

## Acknowledgments

We gratefully acknowledge the vital assistance of Darren Yi Sern Low in providing the Endnote guidance and training.

## Conflict of interest

Author SC’N was employed by company CAIQTEST Malaysia Sdn. Bhd.

The authors declare that this study received funding from MyPower Biotech Sdn. Bhd. (Malaysia). The funder was involved in the study, related to the development of value-added health products from tropical fruits.

## Publisher’s note

All claims expressed in this article are solely those of the authors and do not necessarily represent those of their affiliated organizations, or those of the publisher, the editors and the reviewers. Any product that may be evaluated in this article, or claim that may be made by its manufacturer, is not guaranteed or endorsed by the publisher.
