# Supplementary material for: Essential oils and plant extracts for tropical fruits protection: From farm to table
Source: Front Plant Sci. 2022 Sep 29;13:999270. doi: 10.3389/fpls.2022.999270 (PMC9559231; doi:10.3389/fpls.2022.999270)
Supplement: Supplementary file 1 [file Table_2.pdf]

Supplementary Material

1 Supplementary Table

Supplementary Table 2. Common pathogenic infections in tropical fruit crops.

Fruits	Essential oil/Plant Extract	Form of essential oil/plant extract	Active compound(s)	Study type	Study method	Pathogen/ disease	Reference
Durian	Clove buds extract	Diluted liquid essential oil	Eugenol	*In vitro*	Agar well diffusion method	*P. palmivora*	(Aulifa et al., 2015)
	Clove oil	Pure essential oil vapour	Eugenol	*In vitro*	Disc volatilization method	*P. palmivora*	(Istianto and Emilda, 2021)
	Cosmos caudatus extract	Diluted liquid extract	Sesquiterpene lactones, stigmasterol and lutein	*In vitro*	Agar cup method	*P. palmivora*	(Mohd Salehan et al., 2013)
	Citronella oil Neem oil	Nanoemulsions with Trition-x 100 surfactant	Citronellal, geraniol, citronellal, citral and geranyl acetate	*In vitro*	Poisoned food method	*R. solani*	(Osman Mohamed Ali et al., 2017)
	Hypericum linarioides Bosse extract	Diluted liquid extract	α-Pinene	*In vitro*	Poisoned food method	*R. solani*	(Cakir et al., 2005)
	Cuminum cyminum oil	Diluted liquid essential oil	Pinene, cineone and linalool	*In vitro*	Broth dilution method	*F. solani*	(Naeini et al., 2010)
	Myrcia ovata Cambessedes oil	Diluted liquid essential oil	Linalool, nerolic acid, geraniol, neral, geranial, (E)-	*In vitro*	Poisoned food method	*F. solani*	(Sampaio et al., 2016)

			nerolidol, 1,8-cineole and isopulegol				
	Hydnocarpus anthelminthicus extract	Diluted liquid extract	Flavonoids, isoflavonoids, phenolics, phenol acids, coumarins and alkaloids	*In vitro*	Dilution plate method	*R. solani*<br>*P. palmivora*	(Jantasorn et al., 2016)
	Satureja cuneifolia oil	Diluted liquid essential oil	Camphor and camphene	*In vitro*	Poisoned food method	*P. palmivora*	(Garcia-Rellán et al., 2016)
	Piper chaba Hunter extract	Diluted liquid extract	α-Humulene, caryophyllene oxide, viridiflorol, globulol, β-selinene, spathulenol, (E)-nerolidol, linalool and 3-pentanol	*In vitro*	Poisoned food method	*R. solani*<br>*F. solani*	(Rahman et al., 2011)
	Asarum heterotropoides var. *mandshuricum* essential oil	Diluted liquid essential oil	N.S.	*In vitro*	Poisoned food method	*F. solani*	(Dan et al., 2010)
Banana	*Eucalyptus camaldulensis* extract	Diluted liquid extract	N.S.	*In vitro*	Poisoned food method	*C. gloeosporioides*	(España et al., 2017)
	Basil oil	Diluted liquid essential oil	Eugenol, cinnamyl acetate, humulene, trans-calamenene and caryophyllene	*In vitro*	Poisoned food method, liquid bioassay	*L. theobromae*<br>*C. Musae*<br>*F. proliferatum*	(Anthony et al., 2004)
		Emulsion with Tween 80 surfactant		*In vivo* - preharvest	Preharvest coating	Crown rot	(Siriwardana et al., 2017)
		Pure essential oil vapour		*In vitro*	Disc volatilization method	*C. musae*<br>*L. theobromae*	(Kulkarni et al., 2021)
	Ginger extract	Diluted liquid extract	α-Curcumene and zingerone	*In vitro*	Poisoned food method, cavity slide technique	*C. musae*	(Bhutia et al., 2016)

				In vivo - postharvest	Postharvest coating	Anthracnose	
	Thyme oil	Pure essential oil	Thymol, carvacrol, ρ-cymene and α-pinene	*In vitro*	Poisoned food method	*C. musae*	(Vilaplana et al., 2018a)
				In vivo - postharvest	Postharvest coating	Anthracnose	
	Cinnamon and lemongrass oils	Diluted liquid essential oil	Terpenes, geranial and neral	*In vitro*	Poisoned food method, broth dilution method	*C.musae*<br>*F. incarnatum*<br>*F. verticillioides*	(Kamsu et al., 2019)
	Garlic oil	Incorporated in *Aloe vera* gel	N.S.	*In vitro*	Poisoned food method	*C. musae*	(Khaliq et al., 2019a)
				In vivo - postharvest	Postharvest coating	Anthracnose	
	Garlic extract	Diluted liquid extract	N.S.	*In vivo* - postharvest	Postharvest coating	Crown rot	(Jahan et al., 2019)
	Citronella oil and basil oil	Oil emulsion with sulfonated castor oil surfactant	α-Pinene, citronellol, citronellal, eugenol and geraniol	*In vivo* - postharvest	Postharvest coating	Crown rot	(Anthony et al., 2003)
	Thyme, cinnamon, sweet almond, and bitter almond essential oils	Oil emulsion with Tween 80 surfactant	N.S.	*In vitro*	Poisoned food method	*F. semitectum*	(Abd-Alla et al., 2014)
				In vivo - postharvest	Postharvest coating	Crown rot	
	Zimmu leaf extract	Diluted liquid extract	N.S.	*In vitro*	Poisoned food method	*L. theobromae*<br>*C. musae*	(Sangeetha et al., 2013)
				In vivo - postharvest	Postharvest coating	Crown rot	
	Cinnamon extract	Diluted liquid extract	N.S.	*In vitro*	Poisoned food method	*C. musae*<br>*Fusarium* spp.<br>*L. theobromae*	(Win et al., 2007)
		Diluted liquid extract and in		*In vivo* - postharvest	Postharvest coating	Crown rot	

		combination with chitosan solution					
	Cinnamon oil and clove oil	Diluted liquid essential oil	N.S.	*In vitro*	Broth dilution method	*C. musae*<br>*L. theobromae*<br>*F. proliferatum*	(Ranasinghe et al., 2002)
	Ginger methanolic extract	Diluted liquid extract	α-Curcumin and zingerone	*In vitro*	Poisoned food method	*C. musae*	(Bhutia et al., 2016)
Pineapple	Thyme oil	Diluted liquid extract + Tween 80 surfactants	Thymol, carvacrol, ρ-cymene and α-pinene	*In vitro*	Poisoned food method	F.verticillioides	(Vilaplana et al., 2018b)
				In vivo - postharvest	Postharvest coating	Fusariosis	
	Lemongrass oil	Incorporated into alginate-based coating	Citral	*In vivo* – processed product	Fresh cut fruit coating	N. S.	(Azarakhsh et al., 2014)
	Black pepper and Brazilian pepper essential oil	Diluted liquid essential oil	α-Pinene, limonene, α-phellandrene, 3-carene, β-pinene, sabinene, p-cymene, β-phellandrene and terpinolene	*In vitro*	Broth dilution method	*E. coli*<br>*S. aureus*	(de Araujo et al., 2021)
		Incorporated into alginate-based coating		*In vivo* – processed product	Fresh-cut fruit coating		
	Citrus extract	Diluted liquid extract, with and without benzoate	Terpenes	*In vivo* – processed product	Juice preservatives	*F. oxysporum*	(Bevilacqua et al., 2012)
	Mentha spicata L. essential oil	Diluted liquid essential oil	Carvone and Piperitone oxide	*In vivo* – processed product	Juice preservatives	*P. anomala*<br>*S. cerevisiae*	(da Cruz Almeida et al., 2018)
	Mentha × villosa Huds essential oil	Diluted liquid essential oil	Piperitone oxide	*In vivo* – processed product	Juice preservatives	*S. cerevisiae*	(da Cruz Almeida et al., 2018)
Watermelon	*Azadirachta indica* A. Juss.*, Vitex negundo* Linn and *Persicaria hydropiper* Linn.	Diluted liquid extracts	N.S.	*In vitro*	Dose mortality test	*B. cucurbitae*	(Hossain and Khalequzzaman, 2018)

	Spach methanolic extracts						
	Boerhaavia diffusa root, *Clerodendrum aculeatum* leaf, *Azadirachta indica* leaf and *Terminalia arjuna* bark extracts	Diluted liquid extract	N.S.	*In vivo* - preharvest	Seed treatment and foliar spray	Viral infection	(Sharma et al., 2017)
	Cinnamon oil	Incorporated into alginate-based coating	Cinnamaldehyde	*In vivo* – processed product	Fresh-cut fruit coating	Mould and yeast	(Sipahi et al., 2013)
		Emulsified with Tween 20 surfactants		*In vivo* – processed product	Juice preservatives	*S. typhimurium* *S. aureus*	(Jo et al., 2015)
Papaya	*Aloe vera* gel	Pure gel	N.S.	*In vivo* – postharvest	Postharvest coating	N.S.	(Brishti et al., 2013)
		Diluted gel	N.S.	*In vivo* – postharvest	Postharvest coating	Lesion and soft rot	(Mendy et al., 2019)
	Ginger oil	Incorporated into gum arabic	α-Pinene, 1, 8-cineole and borneol	*In vivo* – postharvest	Postharvest coating	Anthracnose	(Ali et al., 2016)
	Thyme oil and Mexican lime essential oil	Essential oil incorporated into mesquite gum-based edible emulsion	N.S.	*In vivo* – postharvest	Postharvest coating	*C. gloeosporioides* *R. stolonifer*	(Bosquez-Molina et al., 2010)
	Lippia sidoides essential oil	Incorporated into carboxymethyl-cellulose coating	Thymol and carvacrol	*In vivo* – postharvest	Postharvest coating	*C. gloeosporioides*	(Zillo et al., 2018)
	Cinnamon oil	Diluted essential oil incorporated into gum arabic with Tween 80	Cinnamaldehyde	*In vivo* – postharvest	Postharvest coating	Anthracnose	(Maqbool et al., 2011)
				In vitro	Poison food technique and cavity slide technique	*C. gloeosporioides*	

	Lemongrass oil	Incorporated into cassava starch-based coating	N.S.	*In vivo* – processed product	Fresh-cut fruit coating	Mould and yeast	(Praseptiangga et al., 2017)
	Sunflower oil	Refined essential oil incorporated into psyllium gum	N.S.	*In vivo* – processed product	Fresh-cut fruit coating	N.S.	(Yousuf and Srivastava, 2015)
Guava	*Azadirachta indica* A. Juss.*, Vitex negundo* Linn and *Persicaria hydropiper Linn. Spach* methanolic extracts	Diluted liquid extract	N.S.	*In vitro*	Residual film method and sandy-soil method	*B. cucurbitae*	(Hossain and Khalequzzaman, 2018)
	Pomegranate peel extract	Ethanolic extract incorporated into alginate and chitosan coating	N.S.	*In vivo* – postharvest	Postharvest coating	N.S.	(Nair et al., 2018)
	Aloe vera	Pure gel	Pyrocatechol, cinnamic acid and p-coumaric acid	*In vivo* – postharvest, processed product	Postharvest coating, fresh-cut fruit coating	Microbial infection	(Nasution et al., 2015; Kumar et al., 2017)
	Cinnamon and lemongrass oil	Incorporated into arabic gum and sodium caseinate	Geraniol	*In vivo*	Postharvest coating	N.S.	(Murmu and Mishra, 2018)
	Mentha spicata L. essential oil	Emulsion in sterilized Sabouraud dextrose broth with Tween 80 surfactant	Carvone and Piperitone oxide	*In vivo* – processed product	Juice preservatives	*P. anomala*<br>*S. cerevisiae*	(da Cruz Almeida et al., 2018)
	Mentha × villosa Huds essential oils	Emulsion in sterilized Sabouraud dextrose broth with Tween 80 surfactant	Piperitone oxide	*In vivo* – processed product	Juice preservatives	*P. anomala*<br>*S. cerevisiae*	(da Cruz Almeida et al., 2018)

Mangosteen	Virgin coconut oil and cinnamaldehyde	Emulsion	Cinnamaldehyde	*In vitro*	Agar dilution method and Gompertz model	*Glomerella cingulata*	(Permana et al., 2021)
	Citronella oil	Diluted essential oil	Citronellal, citronellol and geraniol	*In vivo* – *pre*harvest	Preharvest coating	Scarring symptom<br>Ants	(Istianto and Emilda, 2021)
	Peppermint oil and lime oil	Essential oil vapour	Limonene, γ -Terpinene, Terpinolene, eucalyptol, menthone, and menthol	*In vivo* – postharvest	Postharvest coating by vapour treatment	N.S.	(Owolabi et al., 2021a; Owolabi et al., 2021b)
Mango	*Mentha piperita* essential oil	Diluted essential oil incorporated into chitosan coating	Menthol and isomenthone	*In vitro*	Poisoned substrate technique	*C. asianum*<br>*C. dianesei*<br>*C. fructicola*<br>*C. tropicale*<br>*C. karstii*	(de Oliveira et al., 2017)
				In vivo – postharvest	Postharvest coating	Anthracnose	
	Ginger oil	Incorporated into hydroxyl propyl methylcellulose (HPMC)	N.S.	*In vivo* – postharvest	Postharvest coating	*C. gloeosporioides*<br>Anthracnose	(Klangmuang and Sothornvit, 2018)
	Galangal essential oil	Incorporated into CMCS and pullulan	N.S.	*In vivo* – postharvest	Postharvest coating	N.S.	(Zhou et al., 2021)
	M. × *villosa* Huds essential oil	Emulsion in sterilized Sabouraud dextrose broth with Tween 80 surfactant	Piperitenone oxide	*In vivo* – precessed products	Juice preservatives	*S. cerevisiae*	(da Cruz Almeida et al., 2018)

Sweet orange	Pomegranate peel extract	Incorporated into chitosan and locust bean gum	Phenolic compounds	*In vivo* – postharvest	Postharvest coating	Green mould	(Kharchoufi et al., 2018)
	Tea tree oil	Incorporated into chitosan coating	N.S.	*In vivo* – postharvest	Postharvest coating	*P. italicum*	(Cháfer et al., 2012)
	Bergamot oil	Incorporated into chitosan coating	N.S.	*In vivo* – postharvest	Postharvest coating	N.S.	
	Thaumatococcus daniellii extract	Diluted liquid extract	N.S.	*In vitro*	Disc diffusion method	*I. orientalis* *M. caribbica* *M. guilliermondii*	(Adeogun et al., 2016)
Rambutan	Wood vinegar and turmeric extract	Crude extract	N.S.	*In vitro*	N.S.	*Oidium nephelii*	(Preecha et al., 2017)
		Crude liquid extract		*In vivo - preharvest*	Preharvest treatment	Powdery mildew	
	Clove oil	Pure liquid essential oil	N.S.	*In vivo - preharvest*	Preharvest treatment	Powdery mildew	(Istianto and Emilda, 2021)
Jackfruit	Vasil (*Ocimum basilicum*) and *Vetiveria zizanioides* essential oils	Diluted liquid essential oil	L-carvone and phenolic compounds	*In vitro*	Disc diffusion method, broth dilution method,	*P. notatum*	(Atif et al., 2020)
				In vivo – postharvest	Postharvest vapour treatment	Postharvest decay	
Dragon fruit	Clove oil	Diluted liquid essential oil	Eugenol	*In vitro*	Broth dilution method, disc diffusion method, microscopy	*A. alternata*	(Castro et al., 2017)
				In vivo – postharvest	Postharvest coating		
	Ginger ethanolic extract	Diluted liquid extract	Gingerol	*In vitro*	Agar dilution method, cavity	*C. gloeosporioides*	(Bordoh et al., 2020)

					slide culture, microscopy		
	Turmeric extract		Curcumin	*In vivo* - postharvest	Postharvest coating	Anthracnose	
	"Dukung anak" extract		Alkaloids	*In vivo* - postharvest	Postharvest coating	Anthracnose	
Salak	Orange essential oil	Pure essential oil vapour	Limonene	*In vivo* – postharvest	Fresh fruit vapour treatment	*Marasmius palmivorus* and *Thieviolopsis* sp.	(Phothisuwan et al., 2021)
Sapodilla	*Fagonia indica* extract	Incorporated into *Aloe vera* gel	N.S.	*In vivo* – postharvest	Postharvest coating	N.S.	(Khaliq et al., 2019b)

References

Abd-Alla, M.A., El-Gamal, N.G., Al-Mougy, N.S., and Abdel-Kader, M.M. (2014). Post-harvest treatments for controlling crown rot disease of Williams banana fruits (*Musa acuminata* L.) in Egypt. *Plant Pathol. Quar.* 4(1)**,** 1-12. doi: 10.5943/ppq/4/1/1.

Adeogun, O., Adekunle, A., and Ashafa, A. (2016). Chemical composition, lethality and antifungal activities of the extracts of leaf of *Thaumatococcus daniellii* against foodborne fungi. *Beni-Suef univ. j. basic appl. sci.* 5(4)**,** 356-368. doi: 10.1016/j.bjbas.2016.11.006.

Ali, A., Hei, G.K., and Keat, Y.W. (2016). Efficacy of ginger oil and extract combined with gum arabic on anthracnose and quality of papaya fruit during cold storage. *J. Food Sci. Technol.* 53(3)**,** 1435-1444. doi: 10.1007/s13197-015-2124-5.

Anthony, S., Abeywickrama, K., Dayananda, R., Wijeratnam, S., and Arambewela, L. (2004). Fungal pathogens associated with banana fruit in Sri Lanka, and their treatment with essential oils. *Mycopathologia* 157(1)**,** 91-97. doi: 10.1023/B:MYCO.0000012226.95628.99.

Anthony, S., Abeywickrama, K., and Wijeratnam, S.W. (2003). The effect of spraying essential oils of *Cymbopogon nardus*, *Cymbopogon flexuosus* and *Ocimum basilicum* on postharvest diseases and storage life of Embul banana. *J. Hortic. Sci. Biotechnol.* 78(6)**,** 780-785. doi: 10.1080/14620316.2003.11511699.

Atif, M., Ilavenil, S., Devanesan, S., AlSalhi, M.S., Choi, K.C., Vijayaraghavan, P., et al. (2020). Essential oils of two medicinal plants and protective properties of jack fruits against the spoilage bacteria and fungi. *Ind. Crops Prod.* 147**,** 112239. doi: 10.1016/j.indcrop.2020.112239.

Aulifa, D., Aryantha, I.N., and Sukrasno, S. (2015). Antifungal *Phytophthora Palmivora* from clove buds (*Syzygium Aromaticum* L.). *Int. J. Pharm. Pharm. Sci.* 7(7)**,** 325-328.

Azarakhsh, N., Osman, A., Ghazali, H.M., Tan, C.P., and Mohd Adzahan, N. (2014). Lemongrass essential oil incorporated into alginate-based edible coating for shelf-life extension and quality retention of fresh-cut pineapple. *Postharvest Biol. Technol.* 88**,** 1-7. doi: 10.1016/j.postharvbio.2013.09.004.

Bevilacqua, A., Campaniello, D., Sinigaglia, M., Ciccarone, C., and Corbo, M.R. (2012). Sodium-benzoate and citrus extract increase the effect of homogenization towards spores of *Fusarium oxysporum* in pineapple juice. *Food Control* 28(2)**,** 199-204. doi: 10.1016/j.foodcont.2012.04.038.

Bhutia, D., Zhimo, V.Y., Kole, R., and Saha, J. (2016). Antifungal activity of plant extracts against *Colletotrichum musae*, the post harvest anthracnose pathogen of banana cv. Martaman. *Nutr. Food Sci.* 46**,** 2-15. doi: 10.1108/NFS-06-2015-0068.

Bordoh, P.K., Ali, A., Dickinson, M., and Siddiqui, Y. (2020). Antimicrobial effect of rhizome and medicinal herb extract in controlling postharvest anthracnose of dragon fruit and their possible phytotoxicity. *Sci. Hortic.* 265**,** 109249. doi: 10.1016/j.scienta.2020.109249.

Bosquez-Molina, E., Jesús, E.R.-d., Bautista-Baños, S., Verde-Calvo, J.R., and Morales-López, J. (2010). Inhibitory effect of essential oils against *Colletotrichum gloeosporioides* and *Rhizopus stolonifer* in stored papaya fruit and their possible application in coatings. *Postharvest Biol. Technol.* 57(2)**,** 132-137. doi: 10.1016/j.postharvbio.2010.03.008.

Brishti, F.H., Misir, J., and Sarker, A. (2013). Effect of biopreservatives on storage life of papaya (*Carica papaya* L.). *Int. J. Food Stud.* 2(1)**,** 126-136. doi: 10.7455/ijfs.v2i1.149.

Cakir, A., Kordali, S., Kilic, H., and Kaya, E. (2005). Antifungal properties of essential oil and crude extracts of *Hypericum linarioides* Bosse. *Biochem. Syst. Ecol.* 33(3)**,** 245-256. doi: 10.1016/j.bse.2004.08.006.

Castro, J.C., Endo, E.H., de Souza, M.R., Zanqueta, E.B., Polonio, J.C., Pamphile, J.A., et al. (2017). Bioactivity of essential oils in the control of *Alternaria alternata* in dragon fruit (*Hylocereus undatus* Haw.). *Ind. Crops Prod.* 97**,** 101-109. doi: 10.1016/j.indcrop.2016.12.007.

Cháfer, M., Sánchez-González, L., González-Martínez, C., and Chiralt, A. (2012). Fungal decay and shelf life of oranges coated with chitosan and bergamot, thyme, and tea tree essential oils. *J. Food Sci.* 77(8)**,** E182-187. doi: 10.1111/j.1750-3841.2012.02827.x.

da Cruz Almeida, E.T., de Medeiros Barbosa, I., Tavares, J.F., Barbosa-Filho, J.M., Magnani, M., and de Souza, E.L. (2018). Inactivation of spoilage yeasts by *Mentha spicata* L. and *M.* × *villosa* Huds. essential oils in cashew, guava, mango, and pineapple Juices. *Front. Microbiol.* 9**,** 1111. doi: 10.3389/fmicb.2018.01111.

Dan, Y., Liu, H.-Y., Gao, W.-W., and Chen, S.-L. (2010). Activities of essential oils from *Asarum heterotropoides* var. *mandshuricum* against five phytopathogens. *Crop Prot.* 29(3)**,** 295-299. doi: 10.1016/j.cropro.2009.12.007.

de Araujo, C.I.M., Bonato, L.B., Mangucci, C.B., Malpass, G.R.P., Okura, M.H., and Granato, A.C. (2021). Comparison of biopolymer-based edible coatings incorporating *Piper nigrum* and *Schinus terebinthifolia* applied on minimally processed pineapple. *Br. Food J.* 124(4)**,** 1274-1284. doi: 10.1108/BFJ-04-2021-0453.

de Oliveira, K.Á.R., Berger, L.R.R., de Araújo, S.A., Câmara, M.P.S., and de Souza, E.L. (2017). Synergistic mixtures of chitosan and *Mentha piperita* L. essential oil to inhibit *Colletotrichum* species and anthracnose development in mango cultivar Tommy Atkins. *Food Microbiol.* 66**,** 96-103. doi: 10.1016/j.fm.2017.04.012.

España, M.D., Arboleda, J.W., Ribeiro, J.A., Abdelnur, P.V., and Guzman, J.D. (2017). Eucalyptus leaf byproduct inhibits the anthracnose-causing fungus *Colletotrichum gloeosporioides*. *Ind. Crops Prod.* 108**,** 793-797. doi: 10.1016/j.indcrop.2017.08.002.

Garcia-Rellán, D., Verdeguer, M., Salamone, A., Blázquez, M.A., and Boira, H. (2016). Chemical composition, herbicidal and antifungal activity of *Satureja cuneifolia* essential oils from Spain. *Nat. Prod. Commun.* 11(6)**,** 1934578X1601100636. doi: 10.1177/1934578X1601100636.

Hossain, S., and Khalequzzaman, M. (2018). Toxicity of three plant leaf extracts against larvae and pupae of melon fruit fly, *Bactrocera cucurbitae* (Coquillett) (Diptera: Tephritidae). *J. Pharmacogn. Phytochem.* 7(2)**,** 3182-3186.

Istianto, M., and Emilda, D. (2021). The potency of citronella oil and clove oil for pest and disease control in tropical fruit plants. *IOP Conference Series: Earth and Environmental Science* 739(1)**,** 012064. doi: 10.1088/1755-1315/739/1/012064.

Jahan, M., Sharmin, R., Chowdhury, M.E.K., Hasan, M., Islam, M., Sikdar, B., et al. (2019). Characterization of crown rot disease of banana fruit and eco-friendly quality improvement approach during storage. *Microbiol. Res. J. Int.* 27**,** 1-13. doi: 10.9734/MRJI/2019/v27i330099.

Jantasorn, A., Moungsrimuangdee, B., and Dethoup, T. (2016). *In vitro* antifungal activity evaluation of five plant extracts against five plant pathogenic fungi causing rice and economic crop diseases. *J. Biopestic.* 9**,** 1-7.

Jo, Y.-J., Chun, J.-Y., Kwon, Y.-J., Min, S.-G., Hong, G.-P., and Choi, M.-J. (2015). Physical and antimicrobial properties of trans-cinnamaldehyde nanoemulsions in water melon juice. *LWT* 60(1)**,** 444-451. doi: 10.1016/j.lwt.2014.09.041.

Kamsu, N.P., Tchinda, S.E., Tchameni, N.S., Jazet, D.P.M., Madjouko, M.A., Youassi Youassi, O., et al. (2019). Antifungal activities of essential oils of cinnamon (*Cinnamomum zeylanicum*) and lemongrass (*Cymbopogon citratus*) on crown rot pathogens of banana. *Indian Phytopathol.* 72(1)**,** 131-137. doi: 10.1007/s42360-018-0104-1.

Khaliq, G., Abbas, H.T., Ali, I., and Waseem, M. (2019a). *Aloe vera* gel enriched with garlic essential oil effectively controls anthracnose disease and maintains postharvest quality of banana fruit during storage. *Hortic. Environ. Biotechnol.* 60(5)**,** 659-669. doi: 10.1007/s13580-019-00159-z.

Khaliq, G., Ramzan, M., and Baloch, A.H. (2019b). Effect of *Aloe vera* gel coating enriched with *Fagonia indica* plant extract on physicochemical and antioxidant activity of sapodilla fruit during postharvest storage. *Food Chem.* 286**,** 346-353. doi: 10.1016/j.foodchem.2019.01.135.

Kharchoufi, S., Parafati, L., Licciardello, F., Muratore, G., Hamdi, M., Cirvilleri, G., et al. (2018). Edible coatings incorporating pomegranate peel extract and biocontrol yeast to reduce *Penicillium digitatum* postharvest decay of oranges. *Food Microbiol.* 74**,** 107-112. doi: 10.1016/j.fm.2018.03.011.

Klangmuang, P., and Sothornvit, R. (2018). Active coating from hydroxypropyl methylcellulose-based nanocomposite incorporated with Thai essential oils on mango (cv. *Namdokmai Sithong*). *Food Biosci.* 23**,** 9-15. doi: 10.1016/j.fbio.2018.02.012.

Kulkarni, S.A., Sellamuthu, P.S., Anitha, D.P.M., and Madhavan, T. (2021). *In vitro* and *in silico* evaluation of antifungal activity of cassia (*Cinnamomum cassia*) and holy basil (*Ocimum tenuiflorum*) essential oils for the control of anthracnose and crown-rot postharvest diseases of banana fruits. *Chem. Pap.* 75(5)**,** 2043-2057. doi: 10.1007/s11696-020-01434-5.

Kumar, A., Singh, O., and Kohli, K. (2017). Post-harvest changes in functional and sensory properties of guava (*Psidium guajava* L. cv. Pant Prabhat) fruits as influenced by different edible coating treatments. *J. Pharmacogn. Phytochem.* 6(6)**,** 1109-1116.

Maqbool, M., Ali, A., Alderson, P.G., Mohamed, M.T.M., Siddiqui, Y., and Zahid, N. (2011). Postharvest application of gum arabic and essential oils for controlling anthracnose and quality of banana and papaya during cold storage. *Postharvest Biol. Technol.* 62(1)**,** 71-76. doi: 10.1016/j.postharvbio.2011.04.002.

Mendy, T.K., Misran, A., Mahmud, T.M.M., and Ismail, S.I. (2019). Application of *Aloe vera* coating delays ripening and extend the shelf life of papaya fruit. *Sci. Hortic.* 246**,** 769-776. doi: 10.1016/j.scienta.2018.11.054.

Mohd Salehan, N., Meon, S., and Ismail, I.S. (2013). Antifungal activity of *Cosmos caudatus* extracts against seven economically important plant pathogens. *Int. J. Agric. Biol.* 15**,** 864-870.

Murmu, S.B., and Mishra, H.N. (2018). The effect of edible coating based on Arabic gum, sodium caseinate and essential oil of cinnamon and lemon grass on guava. *Food Chem.* 245**,** 820-828. doi: 10.1016/j.foodchem.2017.11.104.

Naeini, A., Ziglari, T., Shokri, H., and Khosravi, A.R. (2010). Assessment of growth-inhibiting effect of some plant essential oils on different *Fusarium* isolates. *J. Mycol. Med.* 20(3)**,** 174-178. doi: 10.1016/j.mycmed.2010.05.005.

Nair, M.S., Saxena, A., and Kaur, C. (2018). Effect of chitosan and alginate based coatings enriched with pomegranate peel extract to extend the postharvest quality of guava (*Psidium guajava* L.). *Food Chem.* 240**,** 245-252. doi: 10.1016/j.foodchem.2017.07.122.

Nasution, Z., Ye, J., and Hamzah, Y. (2015). Characteristics of fresh-cut guava coated with *Aloe vera* gel as affected by different additives. *Kasetsart J. (Nat. Sci.)* 49(1)**,** 111-121.

Osman Mohamed Ali, E., Shakil, N.A., Rana, V.S., Sarkar, D.J., Majumder, S., Kaushik, P., et al. (2017). Antifungal activity of nano emulsions of neem and citronella oils against phytopathogenic fungi, *Rhizoctonia solani* and *Sclerotium rolfsii*. *Ind. Crops Prod.* 108**,** 379-387. doi: 10.1016/j.indcrop.2017.06.061.

Owolabi, I.O., Songsamoe, S., Khunjan, K., and Matan, N. (2021a). Effect of tapioca starch coated-rubberwood box incorporated with essential oils on the postharvest ripening and quality control of mangosteen during transportation. *Food Control* 126**,** 108007. doi: 10.1016/j.foodcont.2021.108007.

Owolabi, I.O., Songsamoe, S., and Matan, N. (2021b). Combined impact of peppermint oil and lime oil on mangosteen (*Garcinia Mangostana*) fruit ripening and mold growth using closed system. *Postharvest Biol. Technol.* 175**,** 111488. doi: 10.1016/j.postharvbio.2021.111488.

Permana, A., Sampers, I., and Van der Meeren, P. (2021). Influence of virgin coconut oil on the inhibitory effect of emulsion-based edible coatings containing cinnamaldehyde against the growth of *Colletotrichum gloeosporioides* (*Glomerella cingulata*). *Food Control* 121**,** 107622. doi: 10.1016/j.foodcont.2020.107622.

Phothisuwan, S., Matan, N., and Matan, N. (2021). The influence of a closed system combining orange oil and mode of action on quality preservation of salacca fruit. *Food Control* 130**,** 108265. doi: 10.1016/j.foodcont.2021.108265.

Praseptiangga, D., Utami, R., Khasanah, L. U., Evirananda, I. P., and Kawiji (2017). Effect of cassava starch-based edible coating incorporated with lemongrass essential oil on the quality of papaya MJ9. *IOP Conf. Ser.: Mater. Sci. Eng.* 176, 012054. doi: 10.1088/1757-899X/176/1/012054

Preecha, C., Visuthipath, V., and Sripiak, P. (2017). Sustainable control of powdery mildew (*Pseudoidium nephelii*) of rambutan (*Nephelium lappaceum* Linn.) using medicinal plant crude extracts. *Acta Hortic.* 1178**,** 179-184. doi: 10.17660/ActaHortic.2017.1178.31.

Rahman, A., Al-Reza, S.M., and Kang, S.C. (2011). Antifungal activity of essential oil and extracts of *Piper chaba* Hunter against phytopathogenic fungi. *J. Am. Oil Chem.' Soc* 88(4)**,** 573-579. doi: 10.1007/s11746-010-1698-3.

Ranasinghe, L., Jayawardena, B., and Abeywickrama, K. (2002). Fungicidal activity of essential oils of *Cinnamomum zeylanicum* (L.) and *Syzygium aromaticum* (L.) Merr et L.M. Perry against crown rot and anthracnose pathogens isolated from banana. *Lett. Appl. Microbiol.* 35(3)**,** 208-211. doi: 10.1046/j.1472-765x.2002.01165.x.

Sampaio, T.S., Nizio, D.A.d.C., White, L.A.S., Melo, J.d.O., Almeida, C.S., Alves, M.F., et al. (2016). Chemical diversity of a wild population of *Myrcia ovata* Cambessedes and antifungal activity against *Fusarium solani*. *Ind. Crops Prod.* 86**,** 196-209. doi: 10.1016/j.indcrop.2016.03.042.

Sangeetha, G., Thangavelu, R., Usha Rani, S., and Muthukumar, A. (2013). Antimicrobial activity of medicinal plants and induction of defense related compounds in banana fruits cv. Robusta against crown rot pathogens. *Biol. Control* 64(1)**,** 16-25. doi: 10.1016/j.biocontrol.2011.12.013.

Sharma, N.K., Singh, S., and Awasthi, L.P. (2017). Prevention and control of viral diseases in watermelon through botanical biopesticides. *Virol. Res. Rev.* 1(3)**,** 1-8. doi: 10.15761/VRR.1000114.

Sipahi, R.E., Castell-Perez, M.E., Moreira, R.G., Gomes, C., and Castillo, A. (2013). Improved multilayered antimicrobial alginate-based edible coating extends the shelf life of fresh-cut watermelon (*Citrullus lanatus*). *LWT* 51(1)**,** 9-15. doi: 10.1016/j.lwt.2012.11.013.

Siriwardana, H., Abeywickrama, K., Kannangara, S., Jayawardena, B., and Attanayake, S. (2017). Basil oil plus aluminium sulfate and modified atmosphere packaging controls crown rot disease in Embul banana (*Musa acuminata*, AAB) during cold storage. *Sci. Hortic.* 217**,** 84-91. doi: 10.1016/j.scienta.2017.01.032.

Vilaplana, R., Pazmiño, L., and Valencia-Chamorro, S. (2018a). Control of anthracnose, caused by *Colletotrichum musae*, on postharvest organic banana by thyme oil. *Postharvest Biol. Technol.* 138**,** 56-63. doi: 10.1016/j.postharvbio.2017.12.008.

Vilaplana, R., Pérez-Revelo, K., and Valencia-Chamorro, S. (2018b). Essential oils as an alternative postharvest treatment to control fusariosis, caused by *Fusarium verticillioides*, in fresh pineapples (*Ananas comosus*). *Sci. Hortic.* 238**,** 255-263. doi: 10.1016/j.scienta.2018.04.052.

Win, N.K.K., Jitareerat, P., Kanlayanarat, S., and Sangchote, S. (2007). Effects of cinnamon extract, chitosan coating, hot water treatment and their combinations on crown rot disease and quality of banana fruit. *Postharvest Biol. Technol.* 45(3)**,** 333-340. doi: 10.1016/j.postharvbio.2007.01.020.

Yousuf, B., and Srivastava, A. (2015). Psyllium (*plantago*) gum as an effective edible coating to improve quality and shelf life of fresh-cut papaya (*Carica papaya*). *International Journal of Biological, Biomolecular, Agricultural, Food and Biotechnological Engineering* 9**,** 702-707. doi: 10.5281/zenodo.1107640

Zhou, W., He, Y., Liu, F., Liao, L., Huang, X., Li, R., et al. (2021). Carboxymethyl chitosan-pullulan edible films enriched with galangal essential oil: Characterization and application in mango preservation. *Carbohydr. Polym.* 256**,** 117579. doi: 10.1016/j.carbpol.2020.117579.

Zillo, R.R., da Silva, P.P.M., de Oliveira, J., da Glória, E.M., and Spoto, M.H.F. (2018). Carboxymethylcellulose coating associated with essential oil can increase papaya shelf life. *Sci. Hortic.* 239**,** 70-77. doi: 10.1016/j.scienta.2018.05.025.
